# Familial co-aggregation and shared heritability between depression, anxiety, obesity and substance use

**DOI:** 10.1038/s41398-022-01868-3

**Published:** 2022-03-16

**Authors:** Rujia Wang, Harold Snieder, Catharina A. Hartman

**Affiliations:** 1 Department of Epidemiology, University of Groningen, University Medical Center Groningen, Groningen, Netherlands; 2 Department of Psychiatry, University of Groningen, University Medical Center Groningen, Groningen, Netherlands

**Keywords:** Depression, Addiction

## Abstract

Depression, anxiety, obesity and substance use are heritable and often co-occur. However, the mechanisms underlying this co-occurrence are not fully understood. We estimated their familial aggregation and co-aggregation as well as heritabilities and genetic correlations to improve etiological understanding. Data came from the multi-generational population-based Lifelines Cohort Study (*n* = 162,439). Current depression and anxiety were determined using the MINI International Neuropsychiatric Interview. Smoking, alcohol and drug use were assessed by self-report questionnaires. Body mass index (BMI) and obesity were calculated by measured height and weight. Modified Cox proportional hazards models estimated recurrence risk ratios (λ_R_), and restricted maximum likelihood variance decomposition methods estimated heritabilities (h^2^) and genetic correlations (r_G_). All analyses were adjusted for age, age^2^, and sex. Depression, anxiety, obesity and substance use aggregated within families (λ_R first-degree relative_ = 1.08–2.74) as well as between spouses (λ_R_ = 1.11–6.60). All phenotypes were moderately heritable (from h^2^_depression_ = 0.25 to h^2^_BMI_ = 0.53). Depression, anxiety, obesity and smoking showed positive familial co-aggregation. That is, each of these traits confers increased risk on the other ones within families, consistent with the positive genetic correlations between these phenotypes (r_G_ = 0.16–0.94). The exception was obesity, which showed a negative co-aggregation with alcohol and drug use and vice versa, consistent with the negative genetic correlations of BMI with alcohol (r_G_ = −0.14) and soft drug use (r_G_ = −0.10). Patterns of cross-phenotype recurrence risk highlight the co-occurrence among depression, anxiety, obesity and substance use within families. Patterns of genetic overlap between these phenotypes provide clues to uncovering the mechanisms underlying familial co-aggregation.

## Introduction

Depression, anxiety, obesity and substance use disorders are common diseases, and a major cause of long-term disability and mortality worldwide [1, 2]. The lifetime prevalence of major depressive disorder (MDD) is estimated as 14.6% [3] and that of all anxiety disorders combined add up to 33.7% [4]. There have been large increases in the prevalence of obesity in recent decades, with rates of 36.5% for obesity among adults in the United States (US) [5]. The lifetime prevalence of substance use is also substantial, with estimates of 24.0% for nicotine dependence [6], 17.8% for alcohol abuse [7] and 7.7% for drug abuse [7]. Familial aggregation of these diseases is well-established, with first-degree relatives odds ratios of 2.73 for MDD [8], 3.10 for generalized anxiety disorder (GAD) [8], 2.8–4.6 for obesity [9], 2.13–3.50 for nicotine dependence [10], 2.24 for alcohol abuse/dependence [8] and 2.71 for drug abuse/dependence [8]. Genetic risk explains a substantial part of the familial aggregation. Family and twin studies have shown that many psychiatric and somatic disorders are heritable, with estimated heritabilities of around 37% for MDD [11], 32% for GAD [12], 46% for BMI [13], and 57%, 67% and 61% for alcohol [6], nicotine [6] and drug dependence [14], respectively.

Diseases rarely occur in isolation [15], and depression, anxiety, obesity and substance use often co-occur as well. During the lifespan, three quarters of individuals with MDD develop an anxiety [16], 40% an alcohol disorder [17], 58% nicotine dependence [18], and 10% a drug use disorder [17]. In addition, increased bidirectional risks have been reported, such as between depression and obesity [19]. When these conditions co-occur, consequences may be worse relative to those for each condition alone, such as elevated rate of suicide attempts [20, 21]. Not only do diseases co-occur within the same person but they also co-aggregate within families. Familial co-aggregation has, for example, been shown for MDD and GAD: compared with parents of probands without MDD, parents of probands with MDD have 1.55 times increased risk to develop GAD [8].

Family and twin studies have established that shared genetic risk factors play a key role in the comorbidity across different conditions. The genetic correlation between depression and anxiety is high (r_G_ = 0.70–1.0) (Supplementary Table S1) [22–25]. However, family and twin studies have less often focused on the genetic correlations between other psychiatric disorders and obesity. Evidence of a shared genetic background explaining comorbidity not only comes from family and twin studies but also from recent molecular genetic findings (Supplementary Table S2). A recent genome-wide association meta-analysis identified 44 independent loci for MDD, and two of the loci were in or near loci for obesity and BMI (*OLFM4* and *NEGR1*) [26]. The genetic correlation between MDD and obesity is 0.11–0.20 [26], illustrating the potential contribution of shared genetic risk variants to the comorbidity of MDD and obesity.

Most of familial co-aggregation studies focus on the severe patients using registry data [27], which may cause selection bias and may not be representative of the general population. Besides, in cohort and case-control data the diseases of relatives were usually reported by probands rather than directly measured in relatives [28, 29], which may cause bias. Nonetheless the literature describing familial co-aggregation in the general population is very limited, despite its potential usefulness for early diagnosis and effective prevention of these conditions and their consequences, such as poor health-related quality of life [2], sleep conditions [30, 31], or cardiac diseases [32]. Familial co-aggregation calculated from a large, representative sample of the general population with comprehensive measurements are needed.

Taken together, the objectives of this study are: (1) to estimate familial aggregation and co-aggregation of depression, anxiety, obesity and substance use at the phenotypic level; (2) to quantify the heritability for each phenotype and estimate the strength of overlap between these phenotypes at the genetic level.

## Materials and methods

### Study design and population

Our study was conducted in the ongoing Lifelines Cohort study. Lifelines is a multi-disciplinary prospective population-based cohort study examining in a unique three-generation design the health and health-related behaviours of over 167,000 persons recruited between 2006 and 2013 in the North of The Netherlands [33]. A follow-up visit took place between 2014 and 2017. Lifelines employs a broad range of investigative procedures in assessing the biomedical, socio-demographic, behavioural, physical and psychological factors which contribute to the health and disease of the general population, with a special focus on multi-morbidity and complex genetics. For the current study, we included 162,439 participants aged between 8 and 93 years, who had measurements of depression, anxiety, obesity and substance use at baseline or at the second assessment.

The Lifelines Cohort study is conducted according to the principles of the Declaration of Helsinki and in accordance with the research code of University Medical Center Groningen, and is approved by its medical ethical committee. All participants signed an informed consent form.

### Measurements

#### Depression and anxiety

For adults, current depression and anxiety were measured using the MINI International Neuropsychiatric Interview (MINI) [34]. The MINI was administered as an individual face-to-face interviewed by a trained research nurse at baseline when participants visited a Lifelines research facility. During the follow-up, the MINI was administered as a digital questionnaire, participants entered their answers under the supervision of a trained research nurse on location. Current depression was defined as the presence of major depression measured within the past two weeks or dysthymia within the past two years. Current anxiety was defined as the presence of at least one anxiety disorder, including GAD within the past 6 months, panic disorder within the past month, social anxiety disorder within the past month, and agoraphobia within the past month. The MINI interview at baseline was used to diagnose current depression and anxiety. Initially, in the baseline measurement wave, skips were used in the MINI interview such that some questions were asked, or not asked, depending on the participants’ responses on screening questions. At a later timepoint of the baseline measurement, skips were removed from the MINI. To capture anxiety and depression as a continuous trait using sum scores, we used the MINI without skips at follow-up for participants who had been assessed using the MINI with skips at baseline (*n* = 45,281). We used 10 items in the MINI to calculate sum scores for depression and 10 items for anxiety. For children, depression and anxiety were measured at baseline using children’s behaviour questionnaires [35, 36]. Items corresponding to the fourth edition of the Diagnostic and Statistical Manual of Mental Disorders (DSM-IV) criteria and clinical cut-offs were applied for diagnoses of depression and anxiety for children [37]. Cronbach’s alphas were calculated to estimate internal consistencies of the sum scores of current depression and anxiety. (explained in Supplementary method).

#### Obesity

During the baseline visit, height, weight, waist and hip circumference of participants were measured [33]. BMI was calculated as ${{{\mathrm{BMI}}}} = \frac{{{\rm{Weight}}({\rm{kg}})}}{{{\rm{Height}}^2({\rm{m}}^2)}}$. Waist-hip-ratio (WHR) was calculated as ${{{\mathrm{WHR}}}} = \frac{{{{{\mathrm{Waist}}}}\;{{{\mathrm{circumference}}}}}}{{{\rm{Hip}}\;{{{\mathrm{circumference}}}}}}$. For children, BMI scores were converted to z-scores based on reference data from the 1997 Dutch Growth Study [38]. Obesity was defined as BMI ≥ 30.0 for adults and BMI z-score ≥ 2.0 for children. Overweight was defined as 25.0 ≤ BMI < 30.0 for adults and 1.0 ≤ BMI z-score < 2.0 for children. (explained in Supplementary method).

#### Substance use

Substance use was measured at baseline by questionnaires [39], except for drug use of adults measured at second assessment. Ever smokers were defined as adults who had ever smoked for a full year, and children who had ever smoked during the lifetime. Current smokers were defined as adults who smoked in the past month, and children who smoked in the past 6 months. Cigarettes per day was defined as the number of cigarettes of participants smoked each day. Tobacco high consumption was defined as smoking over 20 cigarettes per day. One packyear was defined as using 20 cigarettes per day for 1 year. For adults, current drinkers were defined as individuals who drank alcohol in the past month. For children, current drinkers were defined as children who drank alcohol in the past 6 months. Daily alcohol intake (grams) was estimated based on the food frequency questionnaire. High alcohol consumption was defined as having a daily alcohol intake over 15 grams. At baseline, drug use was only measured among children (*n* = 8347). Later, drug use was measured among adults at follow-up (*n* = 80,054). Because of a high proportion of loss to follow up of the Lifelines cohort study (35.5%), phenotypes measured at follow-up have a substantial amount of missing data. Drug use was defined as participants having ever used any of the following drugs: cannabis, amphetamines, cocaine, heroin, ecstasy, magic mushrooms and other drugs. Drug use frequency was defined as the number of times of lifetime using any drugs. (explained in Supplementary method).

### Statistical analysis

Continuous data were expressed as mean ± standard deviation, and non-normally distributed data as median and interquartile range. For categorical variables, prevalences were reported. Multilevel logistic regression estimated comorbidity rates across different phenotypes within the same person. Demographic characteristics of participants with complete and with missing data on the phenotypes were compared.

### Recurrence risk ratio

Analyses of familial aggregation of the same phenotype and co-aggregation between different phenotypes were performed for our dichotomous outcome measures using modified Cox proportional hazards model in R3.5.2 (R; Vienna, Austria, 2013) (explained in Supplementary method) [40]. The recurrence risk ratio (λ_R_) was calculated as the ratio between the prevalence of first-degree relatives of participants with the disease under study and its prevalence in the total Lifelines population [41]. In addition to estimating the λ_R_s for first-degree relatives, we estimated λ_R_s for spouses.

### Heritability and genetic correlation

Heritability and genetic correlation were estimated for continuous outcome measures using the Residual Maximum Likelihood-based variance decomposition method in ASReml 4.2 (ASReml; UK, 2016) [42]. Narrow-sense heritability is defined as the proportion of phenotypic variance attributable to additive genetic variance [43], which was calculated as *h*^2^ = *σ*^*2*^_*a*_/(*σ*^2^_*a*_ + *σ*^2^_*e*_), where *σ*^2^_*a*_ is additive genetic variance, and *σ*^2^_*e*_ is the residual variance. In the bivariate analyses, the genetic correlations between two phenotypes were obtained from the estimated additive genetic covariance and variance components as: $r_G = \frac{{\sigma _{A_xA_y}}}{{\sqrt {\sigma _{A_x}^2\sigma _{A_y}^2} }}$, where *σAxA*_*y*_ is the additive genetic covariance between trait *x* and trait *y*, and *σ*^2^*Ax* and *σ*^2^*A*_*y*_ is the additive genetic variance for traits *x* and *y*, respectively (explained in Supplementary method).

For all analyses, a *p* < 0.05 based on two-sided testing was considered statistically significant. All analyses were adjusted for age, age^2^, and sex. Sensitivity analyses were conducted among a subset of the data with the same sample size as for drug use (*n* = 88,401), which was the smallest sample size for studied phenotypes in our paper, to explore statistical power.

## Results

### Demographic characteristics

Among all participants of Lifelines, 162,439 provided information on depression, anxiety, obesity or substance use (detailed in Supplementary Fig. S1). The demographic characteristics of participants are in Table 1. Figure S2 shows the prevalence of depression, anxiety, obesity and substance use at different age groups. Family structure data are in Supplementary Table S3. Missing data analyses indicated that demographic characteristics were comparable for most phenotypes between people with complete and with missing data on the phenotypes. (detailed in Supplementary Tables S4–S5).

**Table 1:** Characteristics of participants in lifelines.

Characteristics	*N*	n/mean ± SD /median(IQR)	Prevalence (%)
Age	162,439	43.18 ± 14.92	
Sex (female)	162,439	94,440	58.14
Ethnicity (white European)	162,370	158,511	97.62
*Obesity*			
Body mass index (BMI)	162,364	25.62 ± 4.63	
Waist-hip-ratio (WHR)	162,360	0.90 ± 0.08	
Overweight	162,364	86,273	53.14
Obesity	162,364	24,418	15.04
*Anxiety*			
Current anxiety	158,094	12,238	7.74
Sum_Anxiety^a^	127,380	0.00 (0.00–2.00)	
Depression			
Current depression	157,567	5,421	3.44
Sum_Depression^a^	127,378	0.00 (0.00–1.00)	
*Smoking*			
Ever-smoker	156,073	79,861	51.17
Current-smoker	156,511	31,496	20.12
Tobacco high consumption	150,112	10,896	7.26
Number of cigarette per day^a^	150,112	0.00 (0.00–10.00)	
Packyears^a^	149,726	0.00 (0.00–8.50)	
*Alcohol use*			
Current drinker	153,269	116,803	76.21
Alcohol high consumption	153,150	23,847	15.57
Daily alcohol intake (g/day)^a^	153,150	3.44 (0.61–10.08)	
*Drug use*^b^			
Drug ever use	88,401	9558	10.81
Soft drug ever use	88,171	9314	10.56
Hard drug ever use	88,386	3207	3.63
Drug use frequency^a^	88,401	0.00 (0.00–0.00)	
Soft drug use frequency^a^	88,171	0.00 (0.00–0.00)	
Hard drug use frequency^a^	88,386	0.00 (0.00–0.00)	

*SD* standard deviation, IQR interquartile range.

Definitions: Sum_Anxiety: sum score of 10 items related to anxiety from MINI, ranged from 0 to 10. Sum_Depression: sum score of 10 items related to depression from MINI, ranged from 0 to 10. Tobacco high consumption: using cigarettes ≥20 per day. 1 packyear: using 20 cigarettes per day for 1 year, or using 1 cigarette per day for 20 years. Alcohol high consumption: daily alcohol intake ≥15 grams per day. For adults, overweight: 25.0 ≤ BMI < 30.0 kg/m^2^; obesity: BMI ≥ 30.0 kg/m^2^; for children, overweight: 1.0 ≤ BMI z-score < 2.0, obesity: BMI z-score ≥ 2.0; the prevalence of overweight in the table include obesity. According to Dutch law, soft drugs include cannabis and magic mushrooms; hard drugs include amphetamine, ecstasy, heroin, cocaine and so on.

^a^Data distribution is left skewed, using median and interquartile range to describe data.

^b^Data on drug use for adults was assessed only in the second assessment, so the sample size of drug use was smaller than other phenotypes measured at baseline.

### Comorbidity

Comorbidity patterns across different phenotypes within same person are in Supplementary Tables S6–S11. High comorbidity was observed between depression and anxiety. Participants with depression or anxiety had higher risks to have obesity, smoke and use drugs, but lower risks to drink alcohol, and vice versa. Obese participants were less likely to drink alcohol or use drugs. With regard to substance use, participants using one substance (i.e. smoking) were at higher risks to use other substances (i.e. alcohol or drugs). All substance users were less likely to be obese.

### Familial aggregation and heritability

**Fig. 1:**
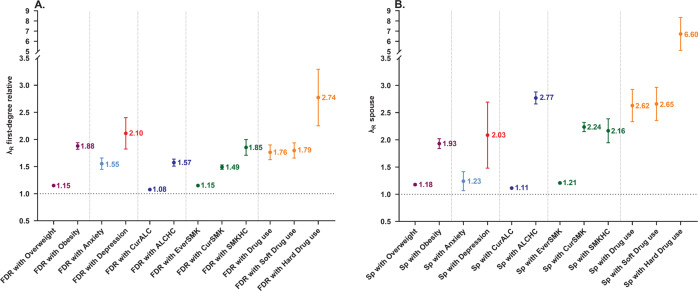
Familial aggregation of obesity, anxiety, depression and substance use. **A** Familial aggregation of obesity, anxiety, depression and substance use among first-degree relatives. **B** Familial aggregation of obesity, anxiety, depression and substance use among spouse. FDR, first-degree relatives; Sp, spouse; CurALC, current drinker; ALCHC, alcohol high consumption: daily alcohol intake ≥15 grams; EverSMK, ever smoker; CurSMK, current smoker; SMKHC, tobacco high consumption: smoking ≥20 cigarettes per day. λ_R_ estimates adjusted for age, age^2^ and sex.

**Fig. 2:**
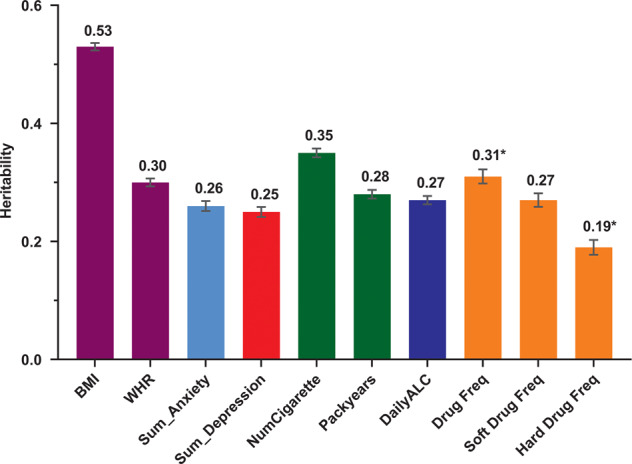
Heritability estimates of obesity, anxiety, depression and substance use. BMI, body mass index; WHR, waist-hip-ratio; Sum_Anxiety, sum score of 10 items related to anxiety from MINI; Sum_Depression, sum score of 10 items related to depression from MINI; NumCigarette, number of cigarettes per day; DailyALC, daily alcohol intake; Drug Freq, lifetime drug use frequency; Soft Drug Freq, lifetime soft drug use frequency; Hard Drug Freq, lifetime hard drug use frequency. Heritability estimates adjusted for age, age^2^ and sex. *Heritabilities for drug use frequency and hard drug use frequency were estimated in the bivariate model with Log converged, because of Log not converged in univariate model.

Findings on familial aggregation analyses are in shown Fig. 1. Participants with a first-degree relative with depression, anxiety, obesity or substance use had higher risks to have the same phenotype (Fig. 1A). The findings indicated that the more severe the phenotype, the higher the first-degree relative recurrence risk ratio (i.e. overweight: 1.15; obese: 1.88). Significant spouse λ_R_s were also found in our study, with the spouse risk comparable with the first-degree relative λ_R_s for depression, anxiety and obesity, and even larger than the first-degree relative λ_R_s for the substance use phenotypes (Fig. 1B). Figure 2 shows the heritabilities of the phenotypes studied. All phenotypes have moderate heritabilities, from 0.25 for depression to 0.53 for BMI.

### Familial co-aggregation

**Fig. 3:**
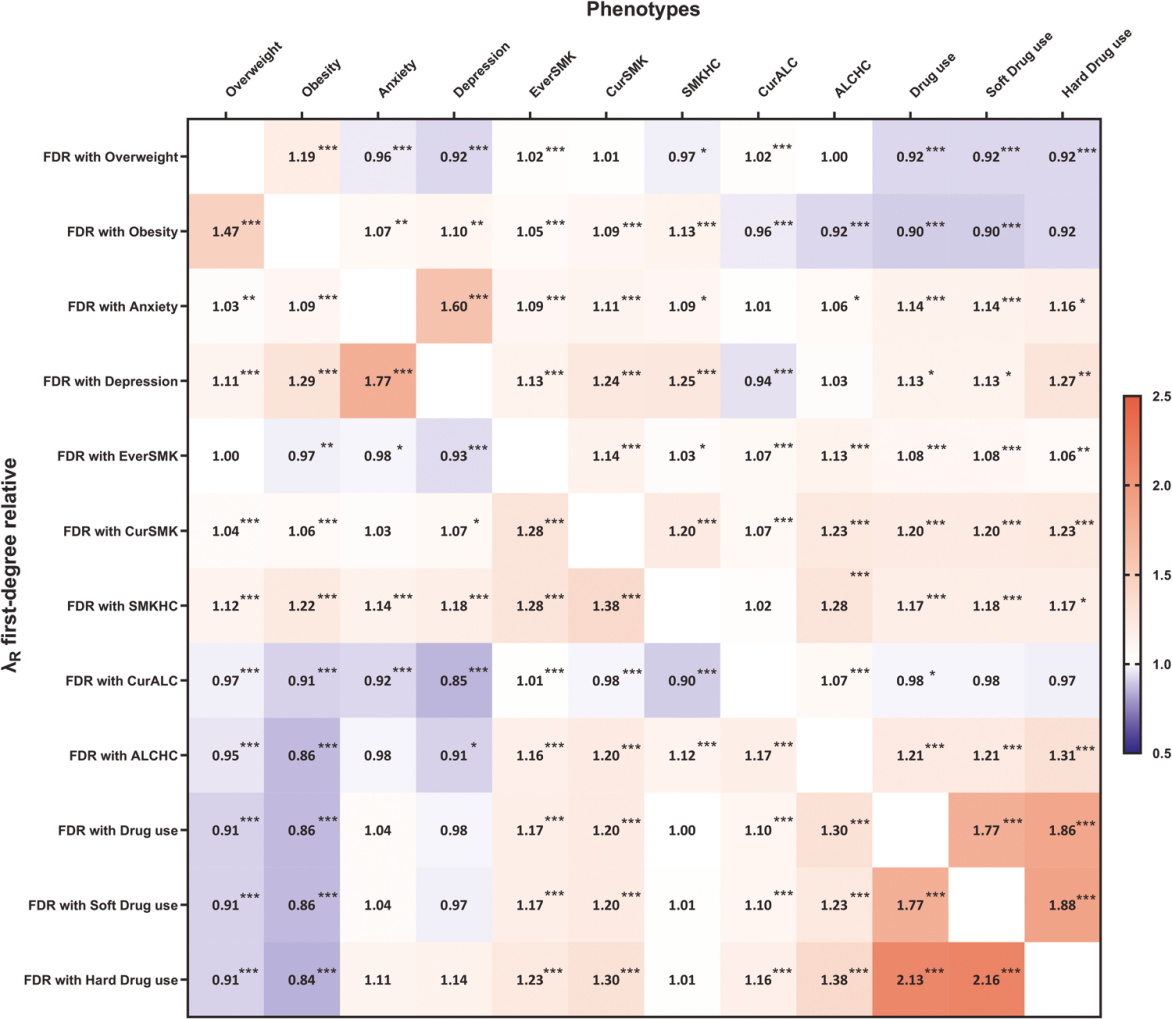
Familial co-aggregation for obesity, anxiety, depression and substance use. FDR, first-degree relative; EverSMK, ever smoker; CurSMK, current smoker; SMKHC, tobacco high consumption: smoking ≥ 20 cigarettes per day; CurALC, current drinker; ALCHC, alcohol high consumption: daily alcohol intake ≥ 15 grams. ^#^λ_R_ adjusted for age, age^2^ and sex. ***p < 0.001, **p < 0.01, *p < *0.05*.

Figure 3 shows the findings on familial co-aggregation analyses expressed as λ_R_s between different phenotypes. Obesity, depression, anxiety, and smoking showed positive familial co-aggregation. That is, each of these traits confers increased risk on the others within families. Besides, depression and anxiety showed positive co-aggregation with drug use, but not with alcohol use. However, alcohol use showed negative co-aggregation, i.e., inverse relationship with depression and anxiety. Likewise, obesity showed negative co-aggregations with alcohol and drug use and vice versa. Smoking, alcohol high consumption, and drug use showed consistent positive co-aggregations within families.

### Genetic, phenotypic and environmental correlations

**Fig. 4:**
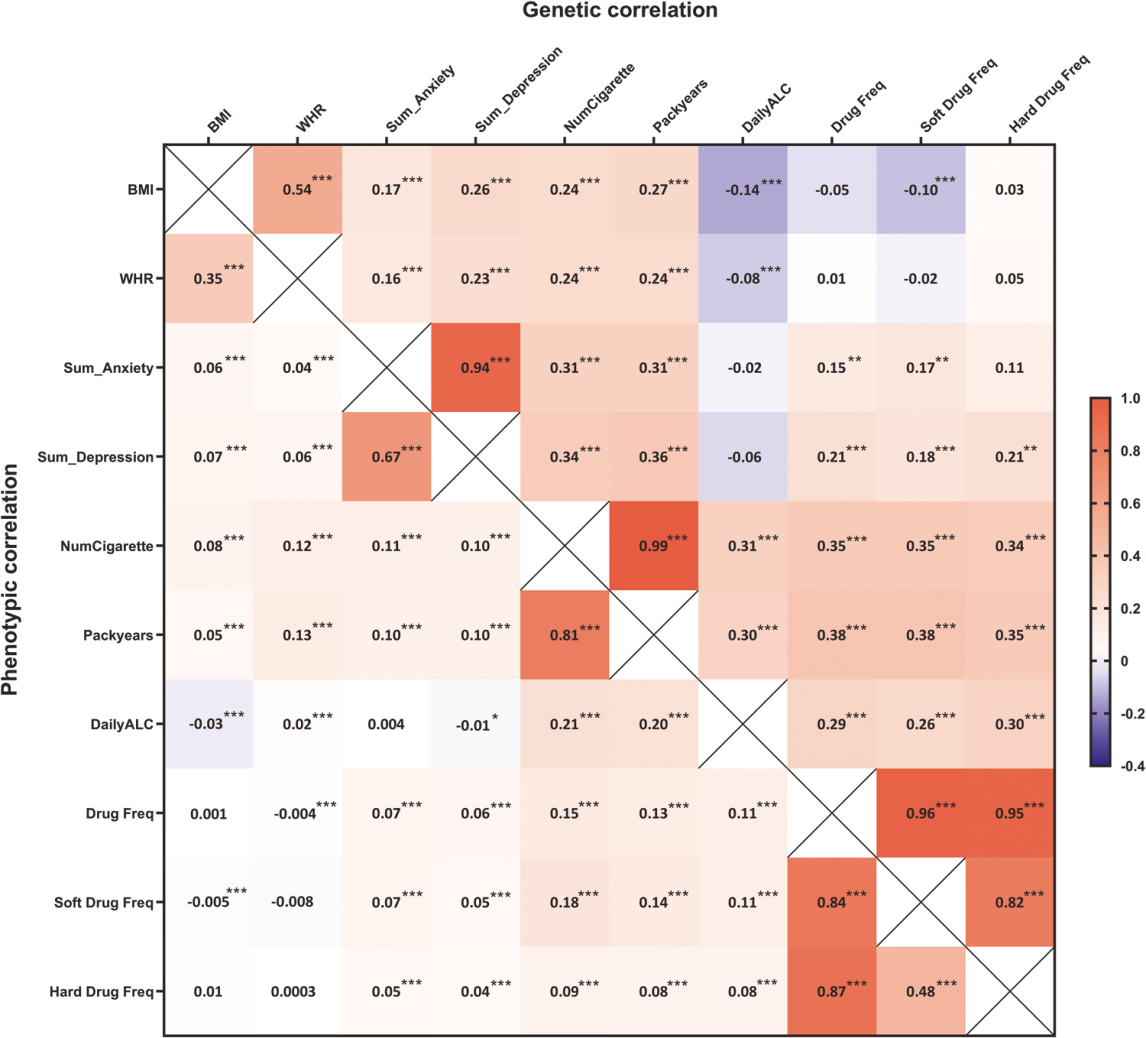
Genetic and phenotypic correlations between obesity, anxiety, depression and substance use. BMI, body mass index; WHR, waist-hip-ratio; Sum_Anxiety, sum score of 10 items related to anxiety from MINI; Sum_Depression, sum score of 10 items related to depression from MINI; NumCigarette, number of cigarettes per day; DailyALC, daily alcohol intake; Drug Freq, lifetime drug use frequency; Soft Drug Freq, lifetime soft drug use frequency; Hard Drug Freq, lifetime hard drug use frequency; r_G_: genetic correlation; r_P_: phenotypic correlation. r_G_ and r_P_ adjusted for age, age^2^ and sex. *P*-value(0): the difference of r_G_ /r_P_ from 0, ***p < 0.001, **p < 0.01, *p < *0.05*. We also test the difference of r_G_ from 1, only r_G_ between Numcigarette and Packyears had non-significant difference from 1.

Genetic and phenotypic correlations are shown in Fig. 4. Genetic correlations between depression, anxiety, obesity and substance use ranged from −0.14 to 0.94. Except for the genetic correlation of depression with anxiety (r_G_ = 0.94), most continuous traits of depression, anxiety, obesity and smoking showed moderate genetic correlations (r_G_ = 0.16–0.36). In addition, BMI had significant negative genetic correlations with daily alcohol intake (r_G_ = −0.14), and soft drug use frequency (r_G_ = −0.10). Compared to the genetic correlations, weaker phenotypic and environmental correlations were generally observed (Supplementary Fig. S3).

Sensitivity analysis among a subset of the data with the same sample size as for drug use (*n* = 88,401) showed that most λ_R_, heritabilities and genetic correlations remained significant and were overall comparable between the total sample and the subsample. Although the smaller sample size had lower statistical power to find associations, there was still ample power to find associations for λ_R_ (7.7% of significant findings lost), heritabilities (0% of significant findings lost) and genetic correlations (0% of significant findings lost). (Supplementary Tables S12–14).

## Discussion

In this large population-based family study, we estimated the familial aggregation and heritability of depression, anxiety, obesity and substance use. Participants with first-degree relatives affected with depression, anxiety, obesity or substance use had a higher risk to have the same condition. Likewise, higher risks were observed among participants with an affected spouse, pointing to shared environmental factors and/or assortative mating. Moreover, our findings reflected the patterns of co-occurrence of depression, anxiety, obesity and substance use within families, and provided estimates of genetic sharing underlying the familial co-occurrence.

Most previous studies have only focused on familial aggregation of subsets of our phenotypes. Our findings are generally consistent with the available evidence from these separate papers. For example, a previous review found a strong association between major depression in the proband and their first-degree relatives (OR = 2.84, 95% CI:2.31–3.49) [11], which converges with the λ_R_ for depression in the present study (λ_R_ = 2.10). In addition, our findings indicated that the more severe the phenotype, the higher the first-degree relatives recurrence risk ratios (e.g. λ_R_ = 1.79 for soft drug use and λ_R_ = 2.74 for hard drug use), convergent with the findings in a Danish study [27]. In this nation-wide registry-based study, individuals with an affected first-degree relative had 3.76-fold higher relative risk for substance use disorder, and 4.54-fold higher relative risk for multiple substance use disorder [27].

Spouses of individuals affected with depression, anxiety, obesity or substance use had substantial higher risks of having the same conditions. As spouses are typically unrelated, the increased risks between spouses may partly be explained by assortative mating (i.e., partner selection based on similarities in certain characteristics) [44]. Based on data from Swedish population registers, a correlation of 0.11 was estimated for the presence of GAD in both partners, 0.12 for depression, and 0.30 for substance abuse [44]. These estimates are consistent with the increased recurrence risks for spouses in the present study (i.e. λ_R_ = 1.23 for anxiety, λ_R_ = 2.03 for depression, λ_R_ = 2.62 for drug use). To the extent that these patterns of spouse resemblance reflect assortative mating, our finding are highly relevant for genetic research. For example, assortative mating violates an important assumption of currently highly popular Mendelian Randomization which may confound conclusions on causality from such studies. Shared environment within families may be another explanation for the increased risks between spouses. For example, an Irish study showed that negative partner interactions were associated with an increased risk for depression and anxiety [45]. As another example, spouse resemblance may reflect the influence of spouses on each other’s health behaviours (e.g. dietary intake, smoking) [46]. Evidence showed that couples-based interventions for health behaviours may be more effective than individual interventions [46], which provides an important angle for early intervention of depression, anxiety, obesity and substance use as well.

The heritability represents the proportion of phenotypic variance that can be attributed to the genetic background. This estimate varies depending on the study population, ethnicity, definition of phenotype, sample size, and study design. A systematic review showed that the heritability of BMI was estimated as 0.75 in twin studies and 0.46 in family studies [13]. A comparable heritability of BMI was found in the current family study (h^2^ = 0.53). Similarly, the heritabilities for depression, anxiety, and substance use estimated in the present study were lower than those estimated in twin studies (h^2^_GAD_: 0.32 [12], h^2^_depression_: 0.37 [11], h^2^_current smoking_: 0.68 [47], h^2^_current alcohol use_: 0.79 [47]). One potential explanation of the consistently lower heritabilities based on family compared to twin studies are that partly different genes may influence the phenotype of interest at different ages [48]. Twins per definition have the same age while this does not hold for pairs of family members in family studies, which often include multiple generations. The age difference in family studies may reduce the correlations (and thereby the heritability estimates) between relatives compared to same aged twins [49]. Heritability estimates in twin studies thus provide an upper bound for amount of phenotypic variance explained by genetic variance. By comparison, single nucleotide polymorphisms (SNPs) based heritability estimates in genome-wide association study (GWAS) usually explain less than half of the heritability estimated in the twin studies (e.g., h^2^_snp_BMI_: 0.19 [50]), as the SNP-based heritability only captures the common genetic variants (i.e. with minor allele frequency >5%). Future research including rare variants (i.e. with minor allele frequency <5%) will likely explain the remaining part of this so called “missing heritability” [51, 52].

We investigated familial co-aggregation and genetic correlations to explore the underlying etiology of co-occurrence between different phenotypes. In the present study, depression and anxiety were significantly co-aggregated within families, and showed the largest genetic overlap (r_G_ = 0.94), confirming findings in previous twin/family and GWAS designs [8, 22, 24, 53]. Only a few family or twin studies have focused on the co-occurrence of psychiatric disorders and obesity. In the present study, depression and obesity were co-aggregated within families, and had a moderate genetic correlation (r_G_ = 0.26). Similar results were reported in a family study in the US, where atypical depression in probands was significantly associated with BMI and overweight in first-degree relatives [54]. In addition, consistent positive genetic correlations of depression and BMI were found in GWAS (r_G_ = 0.09–0.11) [26, 53]. Depression and obesity are both polygenic diseases. Previous studies identified that genes near BMI-associated loci were highly expressed in specific brain regions controlling not only appetite and energy homoeostasis but also mood regulation [55]. In turn, the present study showed that obesity and alcohol or drug use showed negative phenotypic co-aggregation, confirmed by negative genetic correlations. Consistent results were found in a GWAS in the UK Biobank, in which different measures of alcohol intake showed negative genetic correlations with BMI (r_G_ = −0.33 to −0.10) [56]. The inverse genetic correlations might be partly attributable to competition between food and alcohol for similar neurotransmitter receptors (opiate and dopamine receptors), where alcohol intake may affect appetite and metabolism which may alter body composition [57].

In the present study, the substance use phenotypes had variable co-aggregation patterns with depression and anxiety. Smoking and drug use showed positive (i.e., “risk”) co-aggregation patterns for depression and anxiety, while current alcohol use showed inverse relationship with both disorders. The evidence for co-aggregation of risk of depression and anxiety with substance use disorders is overwhelming. A family study from the US observed that alcohol abuse in a first-degree relative resulted in an almost 2-fold higher risk to have MDD or GAD [8]. Similarly, a Danish family study reported that individuals with a first-degree relative affected with substance use disorder had a 1.51-fold and 1.66-fold higher risk to have depression and anxiety, respectively [27]. Moreover, most family studies and GWAS identified consistent positive genetic correlations between substance use disorders and depression or anxiety (r_G_ = 0.30–0.66 in family studies [14, 17], r_G_ = 0.56–0.66 in GWAS [58]). However, it is important to note the distinction between alcohol use on the one hand and abuse and dependence on the other. For familial co-aggregation, our observed protective effect of current alcohol use on depression (and anxiety) was confirmed in a GWAS of the UK Biobank, in which alcohol consumption showed a negative genetic correlation with depressive symptoms (r_G_ = −0.16) [59]. Similar results were found in a Swedish study showing that light drinkers (≤7 drinks/week) and moderate drinkers (7–14 drinks/week) had a lower liability to develop depression, while heavy drinking was associated with a higher liability for depression [60]. Furthermore, alcohol consumption only partly overlapped with alcohol dependence in genetic background (r_G_ = 0.37) [58], indicating that different genetic variants affect alcohol use and alcohol dependence. In addition, alcohol dependence has a strong positive genetic correlations with depression both in family studies and GWAS (r_G_ = 0.56–0.58) [17, 58]. The distributions of the two phenotypes in our sample speak to their differences as well: 76.2% of the participants reported current alcohol use, while only 15.6 % were in the high alcohol use category.

Among the different phenotypes of substance use (smoking, alcohol and drug use), we observed substantial familial co-aggregation and moderate positive genetic correlations (r_G_ = 0.29–0.38). A similar genetic correlation between current smoking and current alcohol use was observed in a Chinese twin study (r_G_ = 0.32) [47]. Many of the genetic variants of substance use phenotypes may be related to neurotransmitter pathways involved in the etiology of addiction [61], which may partly explain the commonly observed comorbidity of different types of substance use.

There are some limitations to the present study. First, depression was measured within the past two weeks, and anxiety was measured within the past six months. As previous studies usually used lifetime depression or anxiety, heritabilities for depression and anxiety might be underestimated due to our definitions of depression and anxiety pertaining to shorter periods. Second, the measurements of some phenotypes were taken at different time points. Lifelines did not measure drug use for adults at baseline. Therefore, we used drug use data for adults measured at follow-up. This resulted in a lower sample size (*n* = 88,401) mainly due to the relatively high proportion of loss to follow up of the Lifelines cohort study (35.5%). Sensitivity analysis among this subset of the data showed that it still provided sufficient power to identify significant familial co-aggregation, heritabilities and genetic correlations. In addition, drug use was defined as lifetime ever use, and was adjusted for age (and age^2^) at the time of data collection. Third, some phenotypes were measured using different questionnaires. The most salient example of this was alcohol use (i.e., alcohol use in 87.62% was measured as part of the food frequency questionnaire while for 12.38% we used slightly differently worded questions). Combining phenotypes based on different measurements increased the sample size, which is why we did this, while at the same time it increases the heterogeneity of the phenotypes. The latter makes interpretation of findings somewhat more complicated. In the specific case of alcohol use, the findings analysed separately for the food frequency questionnaire and the total sample were highly similar. Fourth, affected family members who did not participate in Lifelines may have led to underestimation of our parameters. That is, in general, it is known that more severely affected patients are less likely to participate in population-based studies [62]. However, for Lifelines it has been reported that it is representative of the general population [63]. Finally, 97.6% of the participants in Lifelines were of European ancestry, and our results do therefore not generalize to other ancestries.

In summary, based on our large population-based sample, depression, anxiety, obesity and substance use showed aggregation within families and between spouses, with moderate heritabilities for all phenotypes. In addition, patterns of cross-phenotype recurrence risk highlight the co-occurrence among depression, anxiety, obesity and substance use within families. Awareness of these cross-phenotype patterns may help clinicians diagnose these conditions at an earlier stage, and facilitate timely interventions within families. Genetic overlap between these phenotypes may give clues to uncovering the pleiotropic variants responsible for this. While genetic correlations only give a general indication of the mechanisms, future studies may focus on identifying how genetic variants, potential environmental factors, and interactions between genes and environment explain the co-occurrence of depression, anxiety, obesity and substance use within persons and families.

## Supplementary information

Supplementary material
