# Correction to: Familial co-aggregation and shared heritability between depression, anxiety, obesity and substance use

**DOI:** 10.1038/s41398-022-01906-0

**Published:** 2022-03-30

**Authors:** Rujia Wang, Harold Snieder, Catharina A. Hartman

**Affiliations:** 1 Department of Epidemiology, University of Groningen, University Medical Center Groningen, Groningen, Netherlands; 2 Department of Psychiatry, University of Groningen, University Medical Center Groningen, Groningen, Netherlands

**Keywords:** Depression, Addiction

Correction to: *Translational Psychiatry* 10.1038/s41398-022-01868-3, published online 16 March 2022

The original version of this article unfortunately contained an error in an author name. The correct name is Catharina A. Hartman. The original article has been corrected.

