# Supplementary material for: Familial co-aggregation and shared heritability between depression, anxiety, obesity and substance use
Source: Transl Psychiatry. 2022 Mar 16;12:108. doi: 10.1038/s41398-022-01868-3 (PMC8927111; doi:10.1038/s41398-022-01868-3)
Supplement: Supplementary file 1 — Supplementary material [file 41398_2022_1868_MOESM1_ESM.doc]

**Familial co-aggregation and shared heritability between depression, anxiety, obesity and substance use**

**Supplementary Material**

Part 1: Supplementary Figures

1. Supplementary Fig.S1: Flow chart
2. Supplementary Fig.S2: The prevalence of obesity, anxiety, depression and substance use at different age groups
3. Supplementary Fig.S3: Environmental and phenotypic correlations of obesity, anxiety, depression and substance use

Part 2: Supplementary Table S1-S14

Part 3: Supplementary Methods

**Part 1. Supplementary Figure**

**Fig.S1. Flow chart**


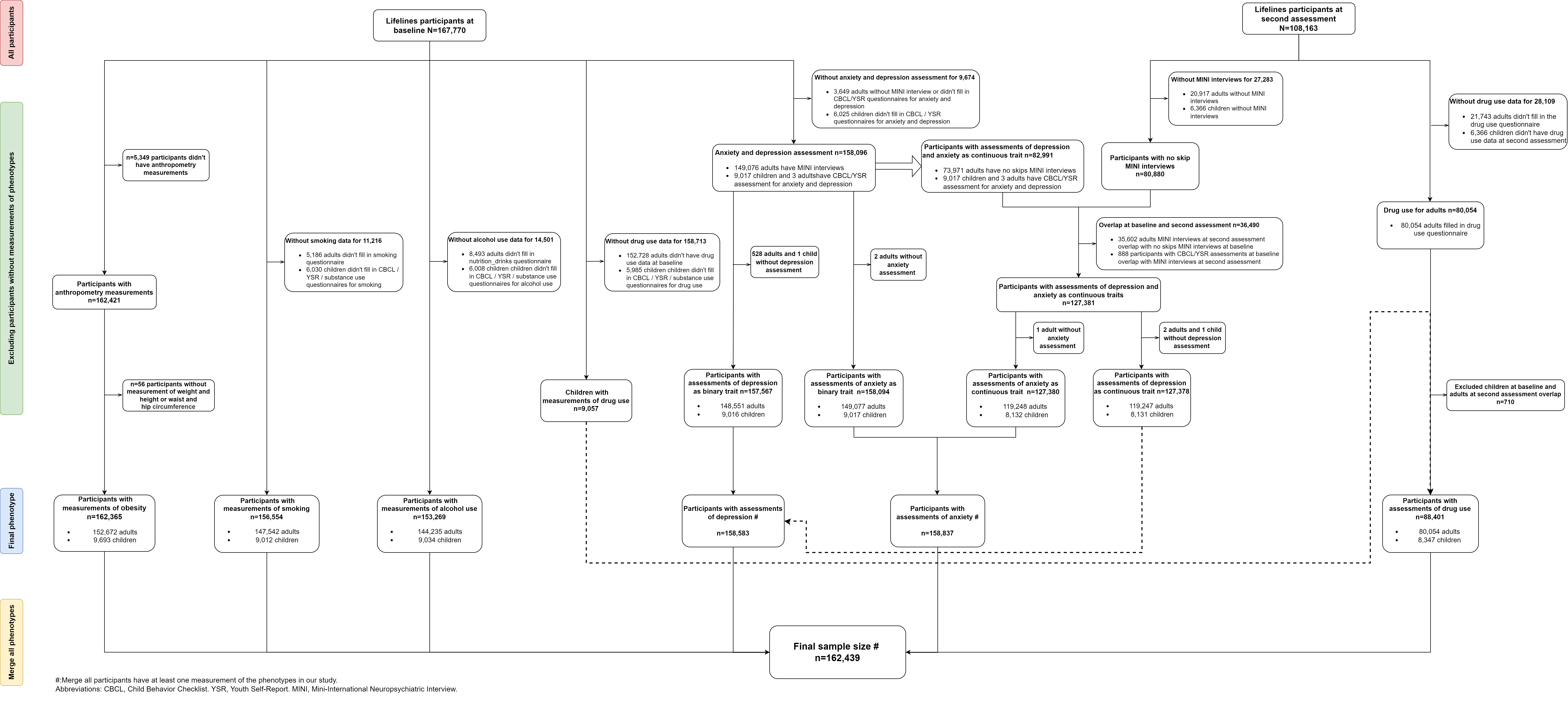


**Fig.S2. The prevalence of obesity, anxiety, depression and substance use at different age groups**





Abbreviations: CurSMK, current smoking; ALCHC, alcohol high consumption: daily alcohol intake ≥15 grams. Error bars indicate 95% confidence interval (CI).

**Fig.S3: Environmental and phenotypic correlations of obesity, anxiety, depression and substance use**





Abbreviations: BMI, body mass index; WHR, waist-hip-ratio; Sum_Anxiety, sum score of 10 items related to anxiety from Mini-international Neuropsychiatric interview (MINI); Sum_Depression, sum score of 10 items related to depression from MINI; NumCigarette, number of cigarettes per day; DailyALC, daily alcohol intake; DrugFreq, lifetime drug use frequency; SoftDrugFreq, lifetime soft drug use frequency; HardDrugFreq, lifetime hard drug use frequency; rE: environmental correlation; rP: phenotypic correlation. rE and rP adjusted for age, age^2^ and sex. P-value(0): the difference of rE /rP from 0, ***: p<0.001, **: p<0.01, *:p<0.05.

**Part 2. Supplementary Table**

| **Table S1. Genetic correlations between depression, anxiety and substance use in twin or family studies** | | | | | | | | | | | | |
| --- | --- | --- | --- | --- | --- | --- | --- | --- | --- | --- | --- | --- |
| Code | Author | PMID | Year | Country | Study design | Sample size | Gender | Trait 1 | Trait 2 | r_G_ | r_E_ | r_P_ |
| 1 | Mosing et al. | 19750555 | 2009 | Australia | Twin study | 5440 twin pairs and 1245 single twin | Male & Female | Depression | Panic disorder | 0.79 | 0.22 | 0.42 |
|  |  |  |  |  |  |  |  | Depression | Agoraphobia | 0.70 | 0.3 | 0.45 |
|  |  |  |  |  |  |  |  | Depression | Social phobia | 0.76 | 0.17 | 0.37 |
| 2 | Kendler et al. | 17121688 | 2007 | Sweden | Twin study | 37296 twins | Male | Major depression | Generalized anxiety disorder | 1.00 | 0.36 | -- |
|  |  |  |  |  |  |  | Female |  |  | 0.74 | 0.59 | -- |
| 3 | Zavos et al. | 19945751 | 2010 | UK | Longitudinal twin study | 2651 adolescents | Male & Female | Depression | Anxiety | 0.77 | 0.48 | 0.62 |
|  |  |  |  |  |  | 1597 adolescents |  | Depression | Anxiety | 0.71 | 0.5 | 0.62 |
| 4 | Guffanti et al. | 27452917 | 2016 | US | high-risk family study | 545 individuals | Male & Female | Major depression | Anxiety | 0.92 | -- | -- |
| 5 | Olvera et al. | 21557468 | 2011 | US | family study | 1122 individuals | Male & Female | Major depression | Alcohol disorders | 0.58 | 0.13 | 0.29 |
|  |  |  |  |  |  |  |  | Major depression | Drug disorders | 0.30 | 0.21 | 0.25 |
| 6 | Hodgson et al. | 27517884 | 2017 | US | family study | 1284 individuals | Male & Female | Major depression | Cannabis use | 0.42 | -0.09 | -- |
| 7 | Hodgson et al. | 27318301 | 2016 | US | family study | 1284 individuals | Male & Female | Anxiety | Alcohol dependence | 0.66 | -- | 0.28 |
|  |  |  |  |  |  |  |  | Anxiety | Drug dependence | 0.62 | -- | 0.41 |
|  |  |  |  |  |  |  |  | Anxiety | Smoking | 0.55 | -- | 0.11 |
| 8 | Zhang et al. | 22853853 | 2012 | China | twin study | 450 twins | Male | Current smoking | Current drinking | 0.32 | -- | -- |
| Abbreviations: rG: genetic correlation; rE: environmental correlation; rP: phenotypic correlation. --: no results available. | | | | | | | | | | | | |

| **Table S2. Genetic correlations between depression, anxiety, obesity and substance use in GWAS studies** | | | | | | | | |
| --- | --- | --- | --- | --- | --- | --- | --- | --- |
| Code | Author | PMID | Year | Sample size | Trait 1 | Trait 2 | r_G_ | *p* |
| 1 | The Brainstorm Consortium | 29930110 | 2018 | 265,218 cases and 784,643 controls | Major depression | Anxiety | 0.79 | 5.97E-07 |
|  |  |  |  |  | Major depression | Ever vs Never smoked | 0.33 | 3.10E-11 |
|  |  |  |  |  | Major depression | Cigarettes smoked per day | 0.12 | 0.16 |
|  |  |  |  |  | Major depression | Body mass index | 0.11 | 5.37E-06 |
|  |  |  |  |  | Depressive symptoms | Anxiety | 0.82 | 5.37E-06 |
|  |  |  |  |  | Depressive symptoms | Ever vs never smoked | 0.25 | 9.50E-06 |
|  |  |  |  |  | Depressive symptoms | Cigarettes smoked per day | 0.27 | 0.003 |
|  |  |  |  |  | Depressive symptoms | Body mass index | 0.13 | 5.85E-05 |
|  |  |  |  |  | Anxiety | Ever vs never smoked | 0.39 | 0.02 |
|  |  |  |  |  | Anxiety | Cigarettes smoked per day | 0.09 | 0.67 |
|  |  |  |  |  | Anxiety | Body mass index | 0.08 | 0.26 |
|  |  |  |  |  | Body mass index | Cigarettes smoked per day | 0.28 | 8.06E-06 |
|  |  |  |  |  | Body mass index | Ever vs never smoked | 0.18 | 1.24E-07 |
| 2 | Howard et al. | 30718901 | 2019 | 807,553 individuals (246,363 depression cases and 561,190 controls) | Depression | Ever vs never smoked | 0.31 | 5.54E-18 |
|  |  |  |  |  | Depression | Cigarettes smoked per day | 0.15 | 0.0121 |
|  |  |  |  |  | Depression | Body mass index | 0.08 | 0.0009 |
|  |  |  |  |  | Depression | Overweight | 0.11 | 0.0001 |
|  |  |  |  |  | Depression | Obesity class 1 | 0.09 | 0.0004 |
|  |  |  |  |  | Depression | Obesity class 2 | 0.08 | 0.0046 |
|  |  |  |  |  | Depression | Obesity class 3 | 0.17 | 0.0003 |
|  |  |  |  |  | Depression | Waist-hip-ratio | 0.12 | 3.56E-07 |
| 3 | Wray et al. | 29700475 | 2018 | 135,458 major depression cases and 344,901 controls | Major depression | Anxiety | 0.8 | 1.40E-08 |
|  |  |  |  |  | Major depression | Ever vs never smoked | 0.29 | 3.50E-14 |
|  |  |  |  |  | Major depression | Cigarettes smoked per day | 0.14 | 2.10E-02 |
|  |  |  |  |  | Major depression | Body mass index | 0.09 | 5.00E-04 |
|  |  |  |  |  | Major depression | Overweight | 0.13 | 1.40E-05 |
|  |  |  |  |  | Major depression | Obesity class 1 | 0.11 | 2.00E-04 |
|  |  |  |  |  | Major depression | Obesity class 2 | 0.12 | 4.00E-04 |
|  |  |  |  |  | Major depression | Obesity class 3 | 0.2 | 2.00E-04 |
|  |  |  |  |  | Major depression | Waist-hip-ratio | 0.12 | 3.00E-05 |
| 4 | Wills et al. | 28238197 | 2017 | 120,890 individuals in UK biobank | drinking alcohol per week | Body mass index | -0.1 | <0.01 |
|  |  |  |  |  | frequency of drinking alcohol | Body mass index | -0.33 | <0.01 |
| 5 | [Clarke et al.](https://www.ncbi.nlm.nih.gov/pubmed/?term=Clarke TK[Author]&cauthor=true&cauthor_uid=28937693) | [28937693](https://www.ncbi.nlm.nih.gov/pubmed/28937693) | 2017 | 112,117 individuals in UK Biobank | Alcohol consumption | Major depression | -0.08 | 0.31 |
|  |  |  |  |  | Alcohol consumption | Depressive symptoms | -0.16 | 4.30E-03 |
|  |  |  |  |  | Alcohol consumption | Ever vs never smoked | 0.4 | 1.44E-10 |
|  |  |  |  |  | Alcohol consumption | Cigarettes smoked per day | -0.09 | 0.31 |
|  |  |  |  |  | Alcohol consumption | Body mass index | -0.15 | 5.00E-04 |
|  |  |  |  |  | Alcohol consumption | Overweight | -0.19 | 5.81E-05 |
|  |  |  |  |  | Alcohol consumption | Obesity class 1 | -0.2 | 2.79E-06 |
|  |  |  |  |  | Alcohol consumption | Obesity class 2 | -0.2 | 3.00E-04 |
|  |  |  |  |  | Alcohol consumption | Obesity class 3 | -0.19 | 6.00E-03 |
|  |  |  |  |  | Alcohol consumption | Waist-hip-ratio | -0.09 | 0.03 |
| 6 | Evangelou et al. | 31358974 | 2019 | 480,842 individuals of European descent | Alcohol consumption | Depressive symptoms | -0.1 | 1.20E-02 |
|  |  |  |  |  | Alcohol consumption | Ever vs never smoked | 0.42 | 1.03E-23 |
|  |  |  |  |  | Alcohol consumption | Body mass index | -0.09 | 1.20E-03 |
|  |  |  |  |  | Alcohol consumption | Overweight | -0.12 | 1.00E-04 |
|  |  |  |  |  | Alcohol consumption | Obesity class 1 | -0.13 | 4.72E-06 |
|  |  |  |  |  | Alcohol consumption | Obesity class 2 | -0.15 | 5.53E-05 |
|  |  |  |  |  | Alcohol consumption | Obesity class 3 | -0.14 | 5.30E-03 |
| 7 | Sanchez-Roige et al. | 30336701 | 2019 | 121,604 individuals in UK Biobank and 20,328 in 23andMe | AUDIT | Major depression | -0.11 | 1.19E-01 |
|  |  |  |  |  | AUDIT | Depressive symptoms | -0.08 | 9.33E-02 |
|  |  |  |  |  | AUDIT | Daily cigarettes | 0.03 | 7.11E-01 |
|  |  |  |  |  | AUDIT | Smoking lifetime use | 0.33 | 3.02E-11 |
|  |  |  |  |  | AUDIT | Cannabis lifetime use | 0.31 | 3.44E-07 |
|  |  |  |  |  | AUDIT | Body mass index | -0.14 | 6.38E-03 |
|  |  |  |  |  | AUDIT | Obesity | -0.16 | 1.09E-05 |
|  |  |  |  |  | AUDIT-C | Major depression | -0.23 | 3.73E-03 |
|  |  |  |  |  | AUDIT-C | Depressive symptoms | -0.19 | 5.57E-05 |
|  |  |  |  |  | AUDIT-C | Daily cigarettes | 0.04 | 6.50E-01 |
|  |  |  |  |  | AUDIT-C | Smoking lifetime use | 0.24 | 1.57E-05 |
|  |  |  |  |  | AUDIT-C | Cannabis lifetime use | 0.26 | 1.13E-04 |
|  |  |  |  |  | AUDIT-C | Body mass index | -0.11 | 4.56E-02 |
|  |  |  |  |  | AUDIT-C | Obesity | -0.17 | 8.61E-06 |
|  |  |  |  |  | AUDIT-P | Major depression | 0.26 | 5.63E-03 |
|  |  |  |  |  | AUDIT-P | Depressive symptoms | 0.3 | 2.98E-08 |
|  |  |  |  |  | AUDIT-P | Daily cigarettes | 0.28 | 3.95E-03 |
|  |  |  |  |  | AUDIT-P | Smoking lifetime use | 0.41 | 1.73E-10 |
|  |  |  |  |  | AUDIT-P | Cannabis lifetime use | 0.46 | 6.77E-08 |
|  |  |  |  |  | AUDIT-P | Body mass index | 0.03 | 6.55E-01 |
|  |  |  |  |  | AUDIT-P | Obesity | 0.01 | 8.98E-01 |
| 8 | Walters et al. | [30482948](https://www.ncbi.nlm.nih.gov/pubmed/30482948) | 2018 | 10,206 alcohol dependence cases and 28,480 controls | Alcohol dependence | Major depression | 0.56 | 3.47E-11 |
|  |  |  |  |  | Alcohol dependence | Depressive symptoms | 0.6 | 2.55E-07 |
|  |  |  |  |  | Alcohol dependence | Cigarettes smoked per day | 0.34 | 5.52E-02 |
|  |  |  |  |  | Alcohol dependence | Nicotine dependence | 0.66 | 2.15E-04 |
|  |  |  |  |  | Alcohol dependence | Smoking initiation | 0.71 | 1.34E-07 |
|  |  |  |  |  | Alcohol dependence | Cannabis initiation | 0.79 | 2.53E-04 |
|  |  |  |  |  | Alcohol dependence | Body mass index | 0.09 | 1.68E-01 |
| 9 | LD_Hub | <http://ldsc.broadinstitute.org/lookup/> | 2020 |  | Depressive symptoms | Overweight | 0.14 | 0.001 |
|  |  |  |  |  | Depressive symptoms | Obesity class 1 | 0.12 | 0.004 |
|  |  |  |  |  | Depressive symptoms | Obesity class 2 | 0.12 | 0.02 |
|  |  |  |  |  | Depressive symptoms | Obesity class 3 | 0.24 | 0.001 |
|  |  |  |  |  | Depressive symptoms | Waist-hip-ratio | 0.18 | 2.80E-05 |
|  |  |  |  |  | Ever vs never smoked | Body mass index | 0.16 | <0.001 |
|  |  |  |  |  | Ever vs never smoked | Overweight | 0.15 | 0.003 |
|  |  |  |  |  | Ever vs never smoked | Obesity class 1 | 0.22 | 4.19E-06 |
|  |  |  |  |  | Ever vs never smoked | Obesity class 2 | 0.17 | 0.003 |
|  |  |  |  |  | Ever vs never smoked | Obesity class 3 | 0.18 | 0.03 |
|  |  |  |  |  | Ever vs never smoked | Waist-hip-ratio | 0.2 | 3.10E-06 |
|  |  |  |  |  | Cigarettes smoked per day | Body mass index | 0.23 | 0.002 |
|  |  |  |  |  | Cigarettes smoked per day | Overweight | 0.34 | <0.001 |
|  |  |  |  |  | Cigarettes smoked per day | Obesity class 1 | 0.33 | <0.001 |
|  |  |  |  |  | Cigarettes smoked per day | Obesity class 2 | 0.32 | 0.001 |
|  |  |  |  |  | Cigarettes smoked per day | Obesity class 3 | 0.29 | 0.03 |
|  |  |  |  |  | Cigarettes smoked per day | Waist-hip-ratio | 0.35 | 1.91E-05 |
| Abbreviations: r_G_: genetic correlation; AUDIT: Alcohol use disorder identification test score; AUDIT-C: Alcohol use disorder identification test score, including item 1-3, focusing on alcohol consumption; AUDIT-P: Alcohol use disorder identification test score, including item 4-10, focusing on the problematic consequences of drinking. | | | | | | | | |

| **Table S3.Type and size of family relationship in Lifelines (N=162,439)** | | |
| --- | --- | --- |
| Relationship | Size* | Percentage^#^ |
| First degree relatives | 103,176 | 0.635 |
| Participants-father | 26,095 | 0.161 |
| Participants-mother | 36,911 | 0.227 |
| Participants-offspring | 45,320 | 0.279 |
| Participants-sibling | 59,336 | 0.365 |
| Participants-spouse | 59,542 | 0.367 |
| *size: the number of participants with the specified family relationship. | |  |
| #percentage: the proportion of participants who have the relevant family relationship. | | |

**Table S4. Comparison of individuals with and without phenotypic data**

**at baseline (N=162,439) on age, sex and ethnicity**

| Phenotypes  defined at baseline | Analysis data | | Missing data | |
| --- | --- | --- | --- | --- |
|  | Sample size | Mean±SD/  percentage | Sample size | Mean±SD/  percentage |
| Obesity | 162,364 | 99.95% | 75 | 0.05% |
| Age |  | 43.18±14.92 |  | 42.83±20.49 |
| Sex(female) |  | 58.14% |  | 53.33% |
| Ethnicity(white European) |  | 97.62% |  | 97.26% |
| Current anxiety | 158,094 | 97.33% | 4,345 | 2.67% |
| Age |  | 43.83±14.49 |  | 53.82±23.67 |
| Sex(female) |  | 58.20% |  | 55.88% |
| Ethnicity(white European) |  | 97.94% |  | 85.99% |
| Current depression | 157,567 | 97.00% | 4,872 | 3.00% |
| Age |  | 42.88±14.50 |  | 52.84±22.88 |
| Sex(female) |  | 58.18% |  | 56.65% |
| Ethnicity(white European) |  | 97.95% |  | 87.04% |
| Smoking |  |  |  |  |
| Current-smoker | 156,511 | 96.35% | 5,928 | 3.65% |
| Age |  | 43.46±14.86 |  | 35.75±14.61 |
| Sex(female) |  | 58.12% |  | 58.69% |
| Ethnicity(white European) |  | 97.68% |  | 96.23% |
| Number of cigarette/day | 150,112 | 92.41% | 12,327 | 7.59% |
| Age |  | 42.95±14.65 |  | 45.92±17.59 |
| Sex(female) |  | 59.05% |  | 47.08% |
| Ethnicity(white European) |  | 97.80% |  | 95.47% |
| Packyears | 149,726 | 92.17% | 12,713 | 7.83% |
| Age |  | 43.11±14.71 |  | 43.96±17.20 |
| Sex(female) |  | 58.32% |  | 55.95% |
| Ethnicity(white European) |  | 97.81% |  | 95.37% |
| Alcohol use | 153,269 | 94.35% | 9,170 | 5.65% |
| Age |  | 43.02±14.55 |  | 45.82±19.93 |
| Sex(female) |  | 58.15% |  | 57.97% |
| Ethnicity(white European) |  | 98.02% |  | 90.92% |

**Table S5. Comparison on age, sex and ethnicity of individuals with missing data on drug use and continuous measures of anxiety and depression, with individuals measured at baseline and at follow-up**

| Phenotypes | Analysis data | | | | | | Missing data | |
| --- | --- | --- | --- | --- | --- | --- | --- | --- |
|  | Total Analysis data | | Analysis data  at baseline | | Analysis data  at follow-up | |  |  |
|  | Sample size | Mean±SD/  percentage | Sample size | Mean±SD/  percentage | Sample size | Mean±SD/  percentage | Sample size | Mean±SD/  percentage |
| Sum_Anxiety | 127,380 | 78.42% | 82,100 | 50.54% | 45,280 | 27.88% | 35,059 | 21.58% |
| Age |  | 42.59±14.85 |  | 41.22±16.12 |  | 43.66±12.06 |  | 45.96±14.96 |
| Sex(female) |  | 58.39% |  | 57.61% |  | 59.79% |  | 57.24% |
| Ethnicity (white European) |  | 98.08% |  | 98.06% |  | 98.13% |  | 95.96% |
| Sum_Depression | 127,378 | 78.42% | 82,101 | 50.54% | 45,277 | 27.87% | 35,061 | 21.58% |
| Age |  | 42.59±14.85 |  | 41.22±16.12 |  | 43.66±12.06 |  | 45.32±14.96 |
| Sex(female) |  | 58.39% |  | 57.61% |  | 59.79% |  | 57.24% |
| Ethnicity (white European) |  | 98.08% |  | 98.05% |  | 98.13% |  | 95.96% |
| Drug use | 88,401 | 54.42% | 8,347 | 5.14% | 80,054 | 49.28% | 74,038 | 45.58% |
| Age |  | 43.49±15.93 |  | 12.32±2.70 |  | 46.74±12.94 |  | 42.81±13.61 |
| Sex(female) |  | 58.59% |  | 51.49% |  | 59.33% |  | 57.60% |
| Ethnicity (white European) |  | 98.38% |  | 97.44% |  | 98.48% |  | 96.72% |

| **Table S6. Multilevel logistic regression for depression** | | | | |
| --- | --- | --- | --- | --- |
| Characteristics | Depression (n=5,421) | No depression (n=152,146) | OR (95%CI)^a^ | p |
| Anxiety | 3,070 (56.6) | 8,959 (5.9) | 20.19 (19.03-21.42) | <0.001 |
| Ever smoker | 3,065 (60.4) | 74,602 (50.8) | 1.57 (1.48-1.66) | <0.001 |
| Current smoker | 1,795 (35.2) | 29,088 (19.7) | 2.16 (2.03-2.29) | <0.001 |
| SMKHC | 762 (15.5) | 10,030 (7.0) | 2.62 (2.42-2.85) | <0.001 |
| Current drinker | 3,249 (64.1) | 112,935 (76.7) | 0.57 (0.53-0.60) | <0.001 |
| ALCHC | 602 (11.9) | 23,105 (15.7) | 0.85 (0.78-0.93) | <0.001 |
| Drug ever use | 405 (16.3) | 9,079 (10.8) | 1.53 (1.36-1.71) | <0.001 |
| Soft drug ever use | 391 (15.8) | 8,851 (10.6) | 1.51 (1.34-1.69) | <0.001 |
| Hard drug ever use | 158 (6.4) | 3,017 (3.6) | 1.76 (1.48-2.08) | <0.001 |
| Obesity | 1,255 (23.2) | 22,100 (14.5) | 1.77 (1.65-1.89) | <0.001 |
| Overweight | 3,109 (57.4) | 80,119 (52.7) | 1.34 (1.26-1.41) | <0.001 |
| ^a^ OR adjusted for age, age^2^ and sex, taking family relationships into account as random effect. Data are presented as n (%). Abbreviations: SMKHC, tobacco high consumption: smoking ≥ 20 cigarettes per day; ALCHC, alcohol high consumption: daily alcohol intake ≥15 grams. | | | | |

| **Table S7. Multilevel logistic regression for anxiety** | | | | |
| --- | --- | --- | --- | --- |
| Characteristics | Anxiety (n=12,238) | No Anxiety (n=145,856) | OR (*95%CI*) ^a^ | p |
| Depression | 3,070 (25.5) | 2,351 (1.6) | 20.17 (19.01-21.41) | <0.001 |
| Ever smoker | 6,937 (59.7) | 71,042 (50.4) | 1.42 (1.36-1.48) | <0.001 |
| Current smoker | 3,450 (29.6) | 27,599 (19.5) | 1.71 (1.64-1.78) | <0.001 |
| SMKHC | 1,392 (12.4) | 9,483 (6.9) | 1.93 (1.81-2.05) | <0.001 |
| Current drinker | 8,240 (71.0) | 108,296 (76.6) | 0.75 (0.72-0.79) | <0.001 |
| ALCHC | 1,645 (14.2) | 22,150 (15.7) | 1.02 (0.96-1.08) | 0.497 |
| Drug ever use | 898 (15.0) | 8,617 (10.7) | 1.58 (1.46-1.71) | <0.001 |
| Soft drug ever use | 878 (14.7) | 8,394 (10.4) | 1.59 (1.47-1.72) | <0.001 |
| Hard drug ever use | 354 (5.9) | 2,841 (3.5) | 1.90 (1.69-2.13) | <0.001 |
| Obesity | 2,416 (19.7) | 21,072 (14.5) | 1.37 (1.31-1.44) | <0.001 |
| Overweight | 6,851 (56.0) | 76,714 (52.6) | 1.17 (1.12-1.21) | <0.001 |
| ^a^ *OR* adjusted for age, age^2^ and sex, taking family relationships into account as random effect. Data are presented as n (%). Abbreviations: SMKHC, tobacco high consumption: smoking ≥ 20 cigarettes per day; ALCHC, alcohol high consumption: daily alcohol intake ≥15 grams. | | | | |

| **Table S8. Multilevel logistic regression for obesity** | | | | |
| --- | --- | --- | --- | --- |
| Characteristics | Obesity (n=24,418) | No obesity (n=137,946) | OR (*95%CI*) ^a^ | p |
| Depression | 1,255 (5.4) | 4,164 (3.1) | 1.76 (1.65-1.88) | <0.001 |
| Anxiety | 2,416 (10.3) | 9,822 (7.3) | 1.37 (1.31-1.44) | <0.001 |
| Ever smoker | 13,190 (56.2) | 66,635 (50.3) | 1.07 (1.04-1.10) | <0.001 |
| Current smoker | 4,446 (18.9) | 27,034 (20.3) | 0.93 (0.90-0.97) | <0.001 |
| SMKHC | 2,679 (12.1) | 8,216 (6.4) | 1.80 (1.72-1.89) | <0.001 |
| Current drinker | 15,886 (70.0) | 100,885 (77.3) | 0.59 (0.57-0.61) | <0.001 |
| ALCHC | 3,185 (14.1) | 20,658 (15.8) | 0.82 (0.78-0.85) | <0.001 |
| Drug ever use | 1,004 (8.2) | 8,548 (11.2) | 0.84 (0.79-0.91) | <0.001 |
| Soft drug ever use | 981 (8.1) | 8,327 (11.0) | 0.85 (0.79-0.91) | <0.001 |
| Hard drug ever use | 298 (2.4) | 2,909 (3.8) | 0.77 (0.68-0.88) | <0.001 |
| ^a^ *OR* adjusted for age, age^2^ and sex, taking family relationships into account as random effect. Data are presented as n (%). Abbreviations: SMKHC, tobacco high consumption: smoking ≥ 20 cigarettes per day; ALCHC, alcohol high consumption: daily alcohol intake ≥15 grams. | | | | |

| **Table S9. Multilevel logistic regression for current smoker** | | | | |
| --- | --- | --- | --- | --- |
| Characteristics | Current smoker (n=31,496) | Non-current smoker (n=125,015) | OR (*95%CI*) ^a^ | p |
| Depression | 1,795 (5.8) | 3,302 (2.7) | 2.17 (2.04-2.30) | <0.001 |
| Anxiety | 3,450 (11.1) | 8,202 (6.7) | 1.70 (1.62-1.77) | <0.001 |
| Current drinker | 26,164 (84.2) | 90,468 (74.2) | 1.71 (1.65-1.77) | <0.001 |
| ALCHC | 8,119 (26.1) | 15,709 (12.9) | 2.48 (2.40-2.56) | <0.001 |
| Drug ever use | 3,674 (24.9) | 5,751 (7.9) | 3.46 (3.30-3.64) | <0.001 |
| Soft drug ever use | 3,615 (24.6) | 5,568 (7.6) | 3.51 (3.34-3.69) | <0.001 |
| Hard drug ever use | 1,469 (10.0) | 1,668 (2.3) | 4.04 (3.75-4.36) | <0.001 |
| Obesity | 4,446 (14.1) | 19,107 (15.3) | 0.92 (0.88-0.95) | <0.001 |
| Overweight | 16,448 (52.2) | 66,996 (53.6) | 0.95 (0.92-0.97) | <0.001 |
| ^a^ *OR* adjusted for age, age^2^ and sex, taking family relationships into account as random effect. Data are presented as n (%). Abbreviations: ALCHC, alcohol high consumption: daily alcohol intake ≥15 grams. | | | | |

| **Table S10. Multilevel logistic regression for current drinker** | | | | |
| --- | --- | --- | --- | --- |
| Characteristics | Current drinker (n=116,803) | Non-current drinker (n=36,466) | OR (*95%CI*) ^a^ | p |
| Depression | 3,249 (2.8) | 1,822 (5.0) | 0.56 (0.53-0.60) | <0.001 |
| Anxiety | 8,240 (7.1) | 3,368 (9.3) | 0.73 (0.69-0.76) | <0.001 |
| Ever smoker | 65,267 (56.1) | 12,712 (35.1) | 1.91 (1.86-1.96) | <0.001 |
| Current smoker | 26,164 (22.4) | 4,900 (13.5) | 1.69 (1.64-1.75) | <0.001 |
| SMKHC | 8,775 (7.8) | 2,121 (5.9) | 0.95 (0.90-1.00) | 0.034 |
| Drug ever use | 8,382 (13.3) | 1,025 (4.4) | 3.98 (3.71-4.28) | <0.001 |
| Soft drug ever use | 8,171 (13.0) | 995 (4.3) | 3.99 (3.71-4.28) | <0.001 |
| Hard drug ever use | 2,836 (4.5) | 296 (1.3) | 4.02 (3.54-4.56) | <0.001 |
| Obesity | 15,886 (13.6) | 6,801 (18.7) | 0.56 (0.55-0.58) | <0.001 |
| Overweight | 62,430 (53.5) | 18,636 (51.1) | 0.74 (0.72-0.76) | <0.001 |
| ^a^ *OR* adjusted for age, age^2^ and sex, taking family relationships into account as random effect. Data are presented as n (%). Abbreviations: SMKHC, tobacco high consumption: smoking ≥ 20 cigarettes per day. | | | | |

| **Table S11. Multilevel logistic regression for drug use** | | | | |
| --- | --- | --- | --- | --- |
| Characteristics | Drug ever use (n=9,558) | Drug never use (n=78,843) | OR (*95%CI*) ^a^ | p |
| Depression | 405 (4.3) | 2,082 (2.7) | 1.63 (1.45-1.82) | <0.001 |
| Anxiety | 898 (9.4) | 5,075 (6.6) | 1.52 (1.40-1.64) | <0.001 |
| Ever smoker | 7,157 (76.0) | 35,913 (46.0) | 5.87 (5.56-6.19) | <0.001 |
| Current smoker | 3,674 (39.0) | 11,055 (14.1) | 3.27 (3.11-3.43) | <0.001 |
| SMKHC | 1,081 (11.8) | 4,378 (5.8) | 2.79 (2.58-3.02) | <0.001 |
| Current drinker | 8,382 (89.1) | 54,853 (71.1) | 3.29 (3.06-3.53) | <0.001 |
| ALCHC | 2,275 (24.2) | 10,607 (13.8) | 2.28 (2.15-2.42) | <0.001 |
| Obesity | 1,004 (10.5) | 11,193 (14.2) | 0.79 (0.73-0.85) | <0.001 |
| Overweight | 4,222 (44.2) | 41,700 (52.9) | 0.80 (0.76-0.84) | <0.001 |
| ^a^ *OR* adjusted for age, age^2^ and sex, taking family relationships into account as random effect. Data are present as n (%). Abbreviations: SMKHC, tobacco high consumption: smoking ≥ 20 cigarettes per day; ALCHC, alcohol high consumption: daily alcohol intake ≥15 grams. | | | | |

**Table S12. Recurrence risk ratios (λ_R_) within and across phenotypes in the total sample and the smallest subsample (available for the drug use phenotype)**

| Phenotype | Family relationship | Total sample (N=162,439) | | Subsample (n=88,401) | |
| --- | --- | --- | --- | --- | --- |
|  |  | *λ_R_* (95%*CI*) | *p* | *λ_R_* (95%*CI*) | *p* |
| Obesity | First degree relatives with Obesity | 1.88 (1.82-1.94) | <2.00e-16 | 1.93 (1.86-2.00) | <2.00e-16 |
|  | Spouse with Obesity | 1.93 (1.84-2.01) | <2.00e-16 | 1.93 (1.82-2.04) | <2.00e-16 |
|  | First degree relatives with Depression | 1.29 (1.20-1.38) | 8.00e-13 | 1.28 (1.17-1.41) | 6.82e-08 |
|  | First degree relatives with Anxiety | 1.09 (1.04-1.14) | 1.53e-04 | 1.09 (1.03-1.16) | 4.19e-03 |
|  | First degree relatives with EverSMK | 0.97 (0.96-0.99) | 2.47e-03 | 0.99 (0.97-1.01) | 0.40 |
|  | First degree relatives with CurSMK | 1.06 (1.03-1.09) | 8.30e-05 | 1.07 (1.03-1.11) | 3.52e-04 |
|  | First degree relatives with SMKHC | 1.22 (1.16-1.28) | 1.44e-15 | 1.28 (1.20-1.36) | 4.85e-14 |
|  | First degree relatives with CurALC | 0.91 (0.90-0.93) | <2.00e-16 | 0.93 (0.91-0.94) | <2.00e-16 |
|  | First degree relatives with ALCHC | 0.86 (0.82-0.90) | 8.22e-11 | 0.84 (0.80-0.88) | 7.31e-13 |
|  | First degree relatives with Drug | 0.86 (0.82-0.91) | 1.05e-07 | 0.91 (0.85-0.97) | 6.16e-03 |
|  | First degree relatives with SoftDrug | 0.86 (0.81-0.91) | 1.51e-07 | 0.90 (0.84-0.97) | 3.19e-03 |
|  | First degree relatives with HardDrug | 0.84 (0.77-0.93) | 3.39e-04 | 0.88 (0.78-0.98) | 0.03 |
|  | First degree relatives with Overweight | 1.19 (1.17-1.21) | <2.00e-16 | 1.22 (1.20-1.24) | <2.00e-16 |
| Overweight | First degree relatives with Overweight | 1.15 (1.14-1.16) | <2.00e-16 | 1.16 (1.15-1.17) | <2.00e-16 |
|  | Spouse with Overweight | 1.18 (1.16-1.19) | <2.00e-16 | 1.17 (1.15-1.18) | <2.00e-16 |
|  | First degree relatives with Depression | 1.11 (1.07-1.15) | 6.61e-08 | 1.13 (1.08-1.18) | 8.34e-07 |
|  | First degree relatives with Anxiety | 1.03 (1.01-1.06) | 6.31e-03 | 1.04 (1.01-1.07) | 0.01 |
|  | First degree relatives with EverSMK | 1.00 (0.99-1.01) | 0.74 | 1.00 (0.99-1.01) | 0.71 |
|  | First degree relatives with CurSMK | 1.04 (1.03-1.06) | 5.49e-09 | 1.05 (1.03-1.07) | 2.54e-07 |
|  | First degree relatives with SMKHC | 1.12 (1.09-1.15) | <2.00e-16 | 1.14 (1.10-1.18) | 1.56e-13 |
|  | First degree relatives with CurALC | 0.97 (0.96-0.98) | <2.00e-16 | 0.98 (0.97-0.99) | 4.49e-08 |
|  | First degree relatives with ALCHC | 0.95 (0.93-0.97) | 8.85e-06 | 0.94 (0.92-0.96) | 3.57e-07 |
|  | First degree relatives with Drug | 0.91 (0.89-0.94) | 4.71e-10 | 0.92 (0.89-0.95) | 2.3e-06 |
|  | First degree relatives with SoftDrug | 0.91 (0.89-0.94) | 3.27e-10 | 0.92 (0.89-0.95) | 2.01e-06 |
|  | First degree relatives with HardDrug | 0.91 (0.87-0.96) | 1.52e-04 | 0.93 (0.87-0.98) | 0.01 |
|  | First degree relatives with Obesity | 1.47 (1.45-1.49) | <2.00e-16 | 1.50 (1.47-1.53) | <2.00e-16 |
| Anxiety | First degree relatives with Anxiety | 1.55 (1.45-1.66) | <2.00e-16 | 1.57 (1.45-1.71) | <2.00e-16 |
|  | Spouse with Anxiety | 1.23 (1.07-1.42) | 4.12e-03 | 1.24 (1.02-1.49) | 0.03 |
|  | First degree relatives with Depression | 1.77 (1.64-1.92) | <2.00e-16 | 1.74 (1.57-1.93) | <2.00e-16 |
|  | First degree relatives with EverSMK | 0.98 (0.95-1.00) | 0.04 | 1.01 (0.98-1.04) | 0.56 |
|  | First degree relatives with CurSMK | 1.03 (0.99-1.07) | 0.15 | 1.05 (1.00-1.11) | 0.06 |
|  | First degree relatives with SMKHC | 1.14 (1.07-1.22) | 8.81e-05 | 1.19 (1.09-1.30) | 1.20e-04 |
|  | First degree relatives with CurALC | 0.92 (0.90-0.94) | <2.00e-16 | 0.95 (0.93-0.98) | 1.30e-04 |
|  | First degree relatives with ALCHC | 0.98 (0.92-1.04) | 0.44 | 0.96 (0.91-1.02) | 0.23 |
|  | First degree relatives with Drug | 1.04 (0.97-1.11) | 0.24 | 1.05 (0.96-1.14) | 0.31 |
|  | First degree relatives with SoftDrug | 1.04 (0.97-1.11) | 0.29 | 1.04 (0.96-1.14) | 0.34 |
|  | First degree relatives with HardDrug | 1.11 (0.99-1.25) | 0.07 | 1.10 (0.94-1.27) | 0.23 |
|  | First degree relatives with Obesity | 1.07 (1.02-1.12) | 4.23e-03 | 1.11 (1.05-1.18) | 4.85e-04 |
|  | First degree relatives with Overweight | 0.96 (0.94-0.98) | 6.44e-04 | 0.98 (0.95-1.01) | 0.20 |
| Depression | First degree relatives with Depression | 2.10 (1.83-2.41) | <2.00e-16 | 2.48 (2.12-2.91) | <2.00e-16 |
|  | Spouse with Depression | 2.03 (1.51-2.72) | 2.61e-06 | 2.08 (1.36-3.17) | 6.56e-04 |
|  | First degree relatives with Anxiety | 1.60 (1.48-1.73) | <2.00e-16 | 1.75 (1.58-1.93) | <2.00e-16 |
|  | First degree relatives with EverSMK | 0.93 (0.90-0.96) | 7.28e-05 | 0.96 (0.92-1.01) | 0.12 |
|  | First degree relatives with CurSMK | 1.07 (1.01-1.30) | 0.02 | 1.11 (1.03-1.20) | 7.25e-03 |
|  | First degree relatives with SMKHC | 1.18 (1.07-1.30) | 9.27e-04 | 1.29 (1.14-1.46) | 7.86e-05 |
|  | First degree relatives with CurALC | 0.85 (0.82-0.87) | <2.00e-16 | 0.90 (0.86-0.93) | 1.07e-08 |
|  | First degree relatives with ALCHC | 0.91 (0.83-0.99) | 0.04 | 0.88 (0.80-0.97) | 0.01 |
|  | First degree relatives with Drug | 0.98 (0.89-1.09) | 0.75 | 1.10 (0.96-1.25) | 0.16 |
|  | First degree relatives with SoftDrug | 0.97 (0.88-1.08) | 0.61 | 1.09 (0.96-1.25) | 0.19 |
|  | First degree relatives with HardDrug | 1.14 (0.96-1.36) | 0.13 | 1.29 (1.04-1.59) | 0.02 |
|  | First degree relatives with Obesity | 1.10 (1.04-1.18) | 2.44e-03 | 1.18 (1.07-1.27) | 3.89e-04 |
|  | First degree relatives with Overweight | 0.92 (0.89-0.95) | 2.98e-06 | 0.97 (0.93-1.02) | 0.17 |
| Ever Smoking | First degree relatives with EverSMK | 1.15 (1.14-1.16) | <2.00e-16 | 1.16 (1.15-1.17) | <2.00e-16 |
|  | Spouse with EverSMK | 1.21 (1.19-1.22) | <2.00e-16 | 1.19 (1.17-1.22) | <2.00e-16 |
|  | First degree relatives with Depression | 1.13 (1.09-1.18) | 9.81e-11 | 1.08 (1.03-1.14) | 1.54e-03 |
|  | First degree relatives with Anxiety | 1.09 (1.06-1.11) | 3.19e-11 | 1.08 (1.05-1.11) | 2.05e-06 |
|  | First degree relatives with CurSMK | 1.28 (1.26-1.30) | <2.00e-16 | 1.27 (1.25-1.30) | <2.00e-16 |
|  | First degree relatives with SMKHC | 1.28 (1.25-1.31) | <2.00e-16 | 1.28 (1.23-1.32) | <2.00e-16 |
|  | First degree relatives with CurALC | 1.01 (1.01-1.02) | 6.78e-04 | 1.02 (1.01-1.03) | 1.27e-05 |
|  | First degree relatives with ALCHC | 1.16 (1.13-1.18) | <2.00e-16 | 1.14 (1.12-1.17) | <2.00e-16 |
|  | First degree relatives with Drug | 1.17 (1.14-1.20) | <2.00e-16 | 1.16 (1.13-1.20) | <2.00e-16 |
|  | First degree relatives with SoftDrug | 1.17 (1.14-1.20) | <2.00e-16 | 1.17 (1.13-1.20) | <2.00e-16 |
|  | First degree relatives with HardDrug | 1.23 (1.17-1.28) | <2.00e-16 | 1.23 (1.17-1.30) | 2.39e-14 |
|  | First degree relatives with Obesity | 1.05 (1.03-1.07) | 5.85e-08 | 1.06 (1.04-1.09) | 1.02e-07 |
|  | First degree relatives with Overweight | 1.02 (1.01-1.03) | 1.77e-04 | 1.02 (1.01-1.03) | 1.21e-04 |
| Current Smoking | First degree relatives with CurSMK | 1.49 (1.45-1.53) | <2.00e-16 | 1.49 (1.44-1.55) | <2.00e-16 |
|  | Spouse with CurSMK | 2.24 (2.15-2.32) | <2.00e-16 | 2.25 (2.14-2.37) | <2.00e-16 |
|  | First degree relatives with Depression | 1.24 (1.17-1.32) | 2.75e-12 | 1.22 (1.12-1.32) | 1.92e-06 |
|  | First degree relatives with Anxiety | 1.11 (1.07-1.16) | 2.83e-07 | 1.11 (1.05-1.17) | 3.40e-04 |
|  | First degree relatives with EverSMK | 1.14 (1.12-1.15) | <2.00e-16 | 1.15 (1.13-1.17) | <2.00e-16 |
|  | First degree relatives with SMKHC | 1.38 (1.32-1.44) | <2.00e-16 | 1.44 (1.36-1.52) | <2.00e-16 |
|  | First degree relatives with CurALC | 0.98 (0.96-0.99) | 1.24e-04 | 0.99 (0.97-1.00) | 0.13 |
|  | First degree relatives with ALCHC | 1.20 (1.15-1.24) | <2.00e-16 | 1.17 (1.13-1.21) | 3.14e-16 |
|  | First degree relatives with Drug | 1.20 (1.15-1.25) | <2.00e-16 | 1.22 (1.15-1.29) | 9.64e-12 |
|  | First degree relatives with SoftDrug | 1.20 (1.15-1.26) | <2.00e-16 | 1.22 (1.15-1.29) | 8.37e-12 |
|  | First degree relatives with HardDrug | 1.30 (1.21-1.40) | 2.20e-12 | 1.35 (1.23-1.49) | 7.07e-10 |
|  | First degree relatives with Obesity | 1.09 (1.06-1.12) | 9.59e-09 | 1.10 (1.06-1.15) | 9.20e-07 |
|  | First degree relatives with Overweight | 1.01 (1.00-1.02) | 0.17 | 1.02 (1.01-1.04) | 0.01 |
| Tobacco High Consumption | First degree relatives with SMKHC | 1.85 (1.71-2.00) | <2.00e-16 | 1.95 (1.78-2.14) | <2.00e-16 |
|  | Spouse with SMKHC | 2.16 (1.95-2.39) | <2.00e-16 | 2.01 (1.76-2.29) | <2.00e-16 |
|  | First degree relatives with Depression | 1.25 (1.13-1.38) | 9.88e-06 | 1.18 (1.03-1.35) | 0.02 |
|  | First degree relatives with Anxiety | 1.09 (1.02-1.17) | 0.01 | 1.10 (1.01-1.21) | 0.03 |
|  | First degree relatives with EverSMK | 1.03 (1.01-1.06) | 0.01 | 1.05 (1.01-1.08) | 7.09e-03 |
|  | First degree relatives with CurSMK | 1.20 (1.15-1.25) | <2.00e-16 | 1.18 (1.12-1.25) | 2.50e-10 |
|  | First degree relatives with CurALC | 0.90 (0.88-0.92) | <2.00e-16 | 0.91 (0.89-0.94) | 3.46e-11 |
|  | First degree relatives with ALCHC | 1.12 (1.05-1.18) | 2.43e-04 | 1.05 (0.98-1.11) | 0.15 |
|  | First degree relatives with Drug | 1.00 (0.93-1.08) | 0.94 | 0.98 (0.90-1.08) | 0.75 |
|  | First degree relatives with SoftDrug | 1.01 (0.93-1.08) | 0.89 | 0.98 (0.89-1.08) | 0.75 |
|  | First degree relatives with HardDrug | 1.01 (0.89-1.15) | 0.87 | 1.02 (0.87-1.20) | 0.81 |
|  | First degree relatives with Obesity | 1.13 (1.07-1.18) | 1.33e-06 | 1.17 (1.10-1.24) | 9.69e-07 |
|  | First degree relatives with Overweight | 0.97 (0.94-0.99) | 0.01 | 0.97 (0.94-1.00) | 0.09 |
| Current alcohol use | First degree relatives with CurALC | 1.08 (1.07-1.08) | <2.00e-16 | 1.07 (1.06-1.08) | <2.00e-16 |
|  | Spouse with CurALC | 1.11 (1.11-1.12) | <2.00e-16 | 1.10 (1.09-1.12) | <2.00e-16 |
|  | First degree relatives with Depression | 0.94 (0.91-0.97) | 9.2e-05 | 0.95 (0.91-0.99) | 0.02 |
|  | First degree relatives with Anxiety | 1.01 (0.99-1.03) | 0.16 | 1.02 (0.99-1.04) | 0.14 |
|  | First degree relatives with EverSMK | 1.07 (1.06-1.08) | <2.00e-16 | 1.07 (1.06-1.08) | <2.00e-16 |
|  | First degree relatives with CurSMK | 1.07 (1.06-1.08) | <2.00e-16 | 1.07 (1.05-1.08) | <2.00e-16 |
|  | First degree relatives with SMKHC | 1.02 (1.00-1.04) | 0.08 | 1.02 (1.00-1.05) | 0.09 |
|  | First degree relatives with ALCHC | 1.17 (1.15-1.19) | <2.00e-16 | 1.16 (1.14-1.18) | <2.00e-16 |
|  | First degree relatives with Drug | 1.10 (1.08-1.13) | <2.00e-16 | 1.10 (1.07-1.12) | 2.38e-14 |
|  | First degree relatives with SoftDrug | 1.10 (1.08-1.13) | <2.00e-16 | 1.10 (1.07-1.12) | 3.90e-14 |
|  | First degree relatives with HardDrug | 1.16 (1.12-1.19) | <2.00e-16 | 1.14 (1.10-1.19) | 5.66e-11 |
|  | First degree relatives with Obesity | 0.96 (0.95-0.98) | 1.02e-06 | 0.97 (0.96-0.99) | 3.07e-03 |
|  | First degree relatives with Overweight | 1.02 (1.01-1.02) | 4.23e-07 | 1.02 (1.01-1.03) | 1.21e-05 |
| Alcohol High Consumption | First degree relatives with ALCHC | 1.57 (1.51-1.64) | <2.00e-16 | 1.49 (1.43-1.55) | <2.00e-16 |
|  | Spouse with ALCHC | 2.77 (2.66-2.88) | <2.00e-16 | 2.75 (2.63-2.88) | <2.00e-16 |
|  | First degree relatives with Depression | 1.03 (0.95-1.11) | 0.46 | 1.03 (0.94-1.14) | 0.49 |
|  | First degree relatives with Anxiety | 1.06 (1.01-1.12) | 0.01 | 1.06 (1.00-1.13) | 0.06 |
|  | First degree relatives with EverSMK | 1.13 (1.11-1.14) | <2.00e-16 | 1.11 (1.09-1.14) | <2.00e-16 |
|  | First degree relatives with CurSMK | 1.23 (1.20-1.27) | <2.00e-16 | 1.20 (1.15-1.24) | <2.00e-16 |
|  | First degree relatives with SMKHC | 1.28 (1.22-1.35) | <2.00e-16 | 1.28 (1.20-1.36) | 1.16e-13 |
|  | First degree relatives with CurALC | 1.07 (1.06-1.08) | <2.00e-16 | 1.05 (1.03-1.07) | 1.18e-09 |
|  | First degree relatives with Drug | 1.23 (1.17-1.29) | <2.00e-16 | 1.22 (1.15-1.29) | 1.46e-11 |
|  | First degree relatives with SoftDrug | 1.23 (1.17-1.29) | <2.00e-16 | 1.23 (1.16-1.30) | 4.77e-12 |
|  | First degree relatives with HardDrug | 1.38 (1.28-1.49) | 1.13e-15 | 1.35 (1.22-1.49) | 2.10e-09 |
|  | First degree relatives with Obesity | 0.92 (0.89-0.95) | 6.35e-06 | 0.91 (0.87-0.95) | 7.7e-05 |
|  | First degree relatives with Overweight | 1.00 (0.98-1.02) | 0.96 | 0.98 (0.96-1.00) | 0.10 |

Abbreviations: EverSMK, ever smoker; CurSMK, current smoker; SMKHC, tobacco high consumption: smoking ≥ 20 cigarettes per day; CurALC, current drinker; ALCHC, alcohol high consumption: daily alcohol intake≥15 grams. λ_R_ adjusted for age, age^2^ and sex.

**Table S13. Heritability in the total sample and the smallest subsample**

**(available for the drug use phenotype)**

| Phenotype | Total sample (N=162,439) | | | Subsample (n=88,401) | | |
| --- | --- | --- | --- | --- | --- | --- |
|  | Sample size | *h^2^ (se)* | *p* | Sample size | *h^2^ (se)* | *p* |
| BMI | 162364 | 0.53 (0.006) | <0.001 | 88363 | 0.54 (0.010) | <0.001 |
| WHR | 162360 | 0.30 (0.007) | <0.001 | 88360 | 0.30 (0.010) | <0.001 |
| Sum_Anxiety | 127380 | 0.26 (0.008) | <0.001 | 78376 | 0.24 (0.012) | <0.001 |
| Sum_Depression | 127378 | 0.25 (0.009) | <0.001 | 78374 | 0.23 (0.012) | <0.001 |
| NumCigarette | 150112 | 0.35 (0.007) | <0.001 | 84516 | 0.35 (0.011) | <0.001 |
| Packyears | 149726 | 0.28 (0.007) | <0.001 | 84581 | 0.31 (0.012) | <0.001 |
| DailyALC | 153150 | 0.27 (0.007) | <0.001 | 86466 | 0.28 (0.011) | <0.001 |

Abbreviations: BMI, body mass index; WHR, waist-hip-ratio; Sum_Anxiety, sum score of 10 items related to anxiety from Mini-international Neuropsychiatric interview (MINI); Sum_Depression, sum score of 10 items related to depression from MINI; NumCigarette, number of cigarettes per day; DailyALC, daily alcohol intake. Heritability adjusted for age, age^2^ and sex.

**Table S14. Genetic correlation between phenotypes in the total sample and the smallest subsample (available for the drug use phenotype)**

| Phenotype1 | Phenotype2 | Total sample (N=162,439) | | | Subsample (n=88,401) | | |
| --- | --- | --- | --- | --- | --- | --- | --- |
|  |  | Sample size | *rG (se)* | *p* | Sample size | *rG (se)* | *p* |
| BMI | WHR | 162359 | 0.54 (0.010) | <0.001 | 88359 | 0.54 (0.015) | <0.001 |
| BMI | Sum_Anxiety | 127340 | 0.17 (0.016) | <0.001 | 78359 | 0.12 (0.023) | <0.001 |
| BMI | Sum_Depression | 127338 | 0.26 (0.017) | <0.001 | 78357 | 0.22 (0.024) | <0.001 |
| BMI | NumCigarette | 150060 | 0.24 (0.013) | <0.001 | 84495 | 0.23 (0.018) | <0.001 |
| BMI | Packyears | 149675 | 0.27 (0.014) | <0.001 | 84560 | 0.27 (0.020) | <0.001 |
| BMI | DailyALC | 153097 | -0.14 (0.014) | <0.001 | 86442 | -0.15 (0.020) | <0.001 |
| WHR | Sum_Anxiety | 127336 | 0.16 (0.021) | <0.001 | 78355 | 0.13 (0.031) | 0.003 |
| WHR | Sum_Depression | 127334 | 0.23 (0.022) | <0.001 | 78353 | 0.20 (0.032) | <0.001 |
| WHR | NumCigarette | 150056 | 0.24 (0.016) | <0.001 | 84492 | 0.25 (0.024) | <0.001 |
| WHR | Packyears | 149671 | 0.24 (0.018) | <0.001 | 84557 | 0.25 (0.025) | <0.001 |
| WHR | DailyALC | 153094 | -0.08 (0.018) | <0.001 | 86439 | -0.08 (0.027) | 0.039 |
| Sum_Anxiety | Sum_Depression | 127377 | 0.94 (0.010) | <0.001 | 78374 | 0.95 (0.016) | <0.001 |
| Sum_Anxiety | NumCigarette | 120360 | 0.31 (0.021) | <0.001 | 75474 | 0.19 (0.030) | <0.001 |
| Sum_Anxiety | Packyears | 120210 | 0.31 (0.024) | <0.001 | 75547 | 0.21 (0.032) | <0.001 |
| Sum_Anxiety | DailyALC | 123495 | -0.02 (0.023) | 0.502 | 77384 | 0.04 (0.033) | 0.399 |
| Sum_Depression | NumCigarette | 120359 | 0.34 (0.022) | <0.001 | 75472 | 0.26 (0.031) | <0.001 |
| Sum_Depression | Packyears | 120209 | 0.36 (0.024) | <0.001 | 75545 | 0.33 (0.033) | <0.001 |
| Sum_Depression | DailyALC | 123493 | -0.06 (0.024) | 0.104 | 77382 | -0.01 (0.035) | 0.888 |
| NumCigarette | Packyears | 147232 | 0.99 (0.004) | <0.001 | 83181 | 0.98 (0.006) | <0.001 |
| NumCigarette | DailyALC | 148532 | 0.31 (0.016) | <0.001 | 83858 | 0.33 (0.025) | <0.001 |
| Packyears | DailyALC | 148146 | 0.30 (0.018) | <0.001 | 83923 | 0.30 (0.026) | <0.001 |

Abbreviations: BMI, body mass index; WHR, waist-hip-ratio; Sum_Anxiety, sum score of 10 items related to anxiety from Mini-international Neuropsychiatric interview (MINI); Sum_Depression, sum score of 10 items related to depression from MINI; NumCigarette, number of cigarettes per day; DailyALC, daily alcohol intake; rG: genetic correlation. rG adjusted for age, age^2^ and sex.

**Part 3: Supplementary Methods**

**Measurement**

**(1) Depression and Anxiety**

For adults, current depression and anxiety were measured using the Mini-international Neuropsychiatric interview (MINI) [1]. The MINI is a brief structured interview for diagnosing psychiatric disorders as defined by the fourth edition of the Diagnostic and Statistical Manual of Mental Disorders (DSM-IV) and the International Classification of Diseases (ICD-10). The items for depression and anxiety from the MINI are listed in Table S15.

**Table S15. Items for depression and anxiety from the MINI**

| Phenotypes | Items | Questions | Answers |
| --- | --- | --- | --- |
| Sum score of depression for adults | A1 | Have you been consistently depressed or down, most of the day, nearly every day, for the past two weeks? | 0=no  1=yes |
|  | A2 | In the past two weeks, have you been much less interested in most things or much less able to enjoy the things you used to enjoy most of the time? |  |
|  | A3A | Was your appetite decreased or increased nearly every day? Did your weight decrease or increase without trying intentionally? |  |
|  | A3B | Did you have trouble sleeping nearly every night (difficulty falling asleep, waking up in the middle of the night, early morning wakening or sleeping excessively)? |  |
|  | A3C | Did you talk or move more slowly than normal or were you fidgety, restless or having trouble sitting still almost every day? |  |
|  | A3D | Did you feel tired or without energy almost every day? |  |
|  | A3E | Did you feel worthless or guilty almost every day? |  |
|  | A3F | Did you have difficulty concentrating or making decisions almost every day? |  |
|  | A3G | Did you repeatedly consider hurting yourself, feel suicidal, or wish that you were dead? |  |
|  | B1 | Have you felt sad, low or depressed most of the time for the last two years? |  |
| Sum score of anxiety for adults | O1A | Have you worried excessively or been anxious about several problems of daily life (problems at work, at home or in your close circle) over the past 6 months? | 0=no  1=yes |
|  | O3A | When you were anxious over the past 6 months, did you, most of the time, feel restless, keyed up or on edge? |  |
|  | O3B | When you were anxious over the past 6 months, did you, most of the time, feel tense? |  |
|  | O3C | When you were anxious over the past 6 months, did you, most of the time, feel tired, weak or exhausted easily? |  |
|  | O3D | When you were anxious over the past 6 months, did you, most of the time, have difficulty concentrating or find your mind going blank? |  |
|  | O3E | When you were anxious over the past 6 months, did you, most of the time, feel irritable? |  |
|  | O3F | When you were anxious over the past 6 months, did you, most of the time, have difficulty sleeping (difficulty falling asleep, waking up in the middle of the night, early morning wakening or sleeping excessively)? |  |
|  | E1 | Have you, on more than one occasion, had spells or attacks when you suddenly felt anxious, frightened, uncomfortable or uneasy, even in situations where most people would not feel that way? |  |
|  | F1 | Do you feel anxious or uneasy in places or situations where you might have a panic attack or the panic-like symptoms we just spoke about, or where help might not be available or escape might be difficult: like being in a crowd, standing in a line (queue), when you are alone away from home or alone at home, or when crossing a bridge, traveling in a bus, train or car? |  |
|  | G1 | In the past month, were you fearful or embarrassed being watched, being the focus of attention, or fearful of being humiliated? This includes things like speaking in public, eating in public or with others, writing while someone watches, or being in social situations. |  |

For children, depression and anxiety were measured with parent-report using the Child Behavior Checklist (CBCL) [2] for ages 8-17 years and self-report using the Youth Self-Report (YSR) [3] for 13-17 years at baseline. Items corresponding to DSM-IV criteria and clinical cut-offs were applied for diagnoses of depression and anxiety as binary traits [4], and sum scores of depression and anxiety were calculated as continuous traits. If either self-report or parent-report clinical diagnosis was “Yes”, the diagnosis of depression (or anxiety) was “Yes”, otherwise, depression (or anxiety) was “No”. For children aged 13-17 years, the sum scores of depression and anxiety were calculated based on self-report items. For those without self-report items, we used parent-report items to calculate their sum score of depression and anxiety. For children aged 8-12 years, the sum scores of depression and anxiety were calculated based on parent-report items. The items for depression and anxiety from the CBCM and the YSR are listed in Table S16.

**Table S16. Items for depression and anxiety from the CBCL and the YSR**

| Phenotypes | Age | Items | Questions | Answers |
| --- | --- | --- | --- | --- |
| Sum score of depression for children | Parent-report CBCL  (8-17 years) | CBCL5 | There are not many things it likes | 0=not at all  1=a little bit or sometimes  2=clearly or often |
|  |  | CBCL14 | Cries a lot |  |
|  |  | CBCL18 | Self-harms, or tries to commit suicide |  |
|  |  | CBCL24 | Does not eat well |  |
|  |  | CBCL35 | Feels useless or inferior |  |
|  |  | CBCL52 | Feels very guilty |  |
|  |  | CBCL54 | Is very tired without reason |  |
|  |  | CBCL76 | Sleeps less than most boys and girls |  |
|  |  | CBCL77 | Sleeps more than most boys and girls |  |
|  |  | CBCL91 | Talks about wanting to kill itself |  |
|  |  | CBCL100 | Problems sleeping |  |
|  |  | CBCL102 | Is not very active, moves slowly or has little energy |  |
|  |  | CBCL103 | Is unhappy, sad or depressed |  |
|  | Self-report YSR  (13-17 years) | ACHYSR5 | There is little that I like |  |
|  |  | ACHYSR14 | I cry a lot |  |
|  |  | ACHYSR18 | I try to deliberately hurt or kill myself |  |
|  |  | ACHYSR24 | I do not eat as well as I should |  |
|  |  | ACHYSR35 | I feel useless or inferior |  |
|  |  | ACHYSR52 | I feel very guilty |  |
|  |  | ACHYSR54 | I feel very tired without knowing why |  |
|  |  | ACHYSR83 | I sleep less than most boys and girls |  |
|  |  | ACHYSR84 | I sleep more than most boys and girls |  |
|  |  | ACHYSR98 | I think about killing myself |  |
|  |  | ACHYSR107 | I have trouble sleeping |  |
|  |  | ACHYSR109 | I don't have a lot of energy |  |
|  |  | ACHYSR110 | I am unhappy, sad or depressed |  |
| Sum score of anxiety for children | Parent-report CBCL  (8-17 years) | CBCL11 | Clings to adults or is too dependent | 0=not at all  1=a little bit or sometimes  2=clearly or often. |
|  |  | CBCL29 | Is afraid of certain animals, situations or locations other than school |  |
|  |  | CBCL30 | Is afraid to go to school |  |
|  |  | CBCL45 | Is nervous, twitchy or tense |  |
|  |  | CBCL50 | Is overly scared or anxious |  |
|  |  | CBCL112 | Worries |  |
|  | Self-report YSR  (13-17 years) | ACHYSR11 | I am too dependent on adults |  |
|  |  | ACHYSR29 | I am afraid of certain animals, situations or locations other than school |  |
|  |  | ACHYSR30 | I am afraid to go to school |  |
|  |  | ACHYSR45 | I am nervous, highly-strung or tense |  |
|  |  | ACHYSR50 | I am too frightened or scared |  |
|  |  | ACHYSR119 | I worry a lot |  |

Cronbach's alphas were calculated to estimate internal consistencies of the sum scores of current depression and anxiety. Results are in Table S17.

**Table S17. Cronbach’s alphas for the sum scores of current depression and anxiety**

|  | Sample size | Percentage | Items | Alpha (95%CI) |
| --- | --- | --- | --- | --- |
| Depression | 127,378 |  |  |  |
| Adults_MINI | 119,247 | 93.62% | 10 | 0.74 (0.73-0.74) |
| Children_CBCL | 5,050 | 3.96% | 13 | 0.68 (0.66-0.70) |
| Children_YSR | 3,081 | 2.42% | 13 | 0.72 (0.70-0.74) |
| Anxiety | 127,380 |  |  |  |
| Adults_MINI | 119,248 | 93.62% | 10 | 0.84 (0.83-0.84) |
| Children_CBCL | 5,051 | 3.96% | 6 | 0.68 (0.66-0.70) |
| Children_YSR | 3,081 | 2.42% | 6 | 0.60 (0.57-0.62) |

**(2) Obesity**

At baseline, participants aged 8 years and older were invited to one of twelve Lifelines Research sites for a physical examination by a trained research nurse [5]. During this baseline visit, height without shoes was measured with a SECA 222 stadiometer and rounded to the nearest 0.5 cm. Weight without shoes and heavy clothing was measured with a SECA 761 scale and rounded to the nearest 0.1 kg. Waist and hip circumference were measured with a SECA 200 measuring tape and rounded to the nearest 0.5 cm.

According to the standard international classification of the World Health Organization (WHO), juvenile participants aged 8 to 18 years were classified into 4 BMI z-score classes: underweight (BMI z-score<-2.0), normal weight (-2.0≤ BMI z-score<1.0), overweight (1.0≤BMI z-score<2.0), obesity (BMI z-score≥2.0). Likewise, adults participants were classified into 4 BMI classes: underweight (BMI<18.5), normal weight (18.5≤ BMI<25.0), overweight (25.0 ≤ BMI<30.0), and obesity (BMI ≥ 30.0). To estimate the recurrence risk ratio, we combined 4 BMI classes into 2 classes to generate binary overweight (underweight or normal weight vs overweight including obesity) and binary obesity (underweight or normal weight or overweight vs obesity) in our analysis.

**(3) Smoking**

For adults, smoking was measured by a self-report questionnaire [6] at baseline. Current smoking was defined using the question “Do you smoke now, or have you smoked in the past month? (Yes/No)” Ever smoking was defined using the question “Have you ever smoked for a full year? (Yes/No)” Number of cigarettes per day was defined using the question “How much cigarettes and/or roll-ups do you smoke on average a day now?” Packyears were calculated by the formula: Packyears = number of cigarettes per day/20 *duration of smoking (years).

For children aged 13-17 years, smoking was measured at baseline by parent-report using the CBCL [2], and self-report using the YSR [3]. Current smoking was defined by combining the YSR self-report question “I smoked tobacco now or in the past 6 months” and the CBCL parent-report question “To what extent does this description apply to your child now or in the past 6 months: smokes tobacco?” If one of the answer is “Yes”, current smoking was coded as “Yes”, otherwise, it was coded as “No”. Ever smoking was defined by combining the self-report question “Have you ever smoked cigarettes or roll-ups, even if that was only one cigarette or a couple of puffs?” and the response to current smoking (Yes/No). Number of cigarettes per day was defined by combining the self-report question “On a day that you smoke, how many cigarettes/roll-ups do you smoke?” and parent-report question “How many cigarettes does/did your child smoke every day on average?” Comparing the results of these two questions, we chose the larger number of reported cigarettes used per day. Packyears were calculated by the same formula as for adults.

For children aged 8-12 years, current smoking was defined using the parent-report CBCL question “To what extent does this description apply to your child now or in the past 6 months: smokes tobacco?” If the answer was “a little bit or sometimes” or “clearly or often”, current smoking was coded as “Yes”, otherwise it was coded as “No”. Ever smoking was coded as “Yes” when a child was a current smoker, otherwise it was coded as “No”. As there was no direct question to measure the number of cigarettes per day for children aged 8-12 years, and given the young age, we used 0.5 cigarette per day as a conservative estimate for current smokers (n=1).

**(4) Alcohol use**

For adults, daily alcohol intake (grams) was estimated based on a food frequency questionnaire (FFQ) developed by Wageningen University (WUR) [7]. Current drinking was defined using the question “How often did you drink alcoholic drinks in the past month?” If the answer was “Not this month”, current drinking was “No”, and if otherwise, “Yes”.

For adults recruited for the baseline visit between March 2009 and December 2013, daily alcohol intake was calculated by a formula developed by WUR: Daily alcohol intake (grams) = frequency * amount (glass) * weighted (different type of drinks) * alcohol/glass. (1) Frequency was “never”=0, “1 day a month”=1/28, “2-3 days a month”=2.5/28, “1 day a week”=1/7, “2-3 days a week”=2.5/7, “4-5 days a week”=4.5/7, “6-7 days a week”=6.5/7. (2) If someone drank different kinds of alcohol, the amounts of the different types of alcohol were weighted: “never”=0%, “sometimes”=33%, “often”=67% and “always”=100%. According to the “Dutch dietary guidelines 2015”, a standard glass of alcoholic drink is defined as containing roughly 10 grams of alcohol in the Netherlands. Table S18 shows the amount of alcohol in different types of alcoholic beverages. Table S19 shows the items for alcohol use from the FFQ.

**Table S18. The amount of alcohol in different types of alcoholic beverages**

| Alcoholic beverages | Amount | Alcohol (grams) |
| --- | --- | --- |
| Beer | 1 glass | 8.84 |
| Beer | 1 bottle/can (330ml) | 12.69 |
| Beer | 1 swing top cap bottle/pint (500ml) | 19.23 |
| Alcohol-free beer | 1 glass | 0.10 |
| Red wine | 1 glass | 10.01 |
| White wine | 1 glass | 9.10 |
| Sherry/port/vermouth/madeira | 1 glass | 6.80 |
| Distilled wine | 1 glass | 9.67 |
| Other alcoholic beverages | 1 glass | 6.80 |

**Table S19. The items for alcohol use in the FFQ**

| Items | Questions | Answers |
| --- | --- | --- |
| Food27 | How often did you drink alcoholic drinks in the past month? This includes non-alcoholic beer | “never”=0  “1 day a month”=1/28,  “2-3 days a month”=2.5/28,  “1 day a week”=1/7,  “2-3 days a week”=2.5/7, “4-5 days a week”=4.5/7, “6-7 days a week”=6.5/7. |
| Food27A | How many glasses (of alcoholic drinks) did you drink per day on average? |  |
| Food28D | Which types of alcoholic drink did you drink? Non-alcoholic beer | “never”=0%, “sometimes”=33%, “often”=67% ,  “always”=100%. |
| Food28E | Which types of alcoholic drink did you drink? Beer |
| Food32A1 | Which types of alcoholic drink did you drink? Red wine |
| Food32A2 | Which types of alcoholic drink did you drink? White wine |
| Food32A3 | Which types of alcoholic drink did you drink? Rose wine |
| Food33A | Which types of alcoholic drink did you drink? Sherry, port, vermouth, Madeira |
| Food33A | Which types of alcoholic drink did you drink? Spirits (such as gin, rum, Dutch brandy, whisky, Jägermeister, cognac, brandy) |
| Food35 | Which types of alcoholic drink did you drink? Other types of alcoholic drink |

For adults recruited for the baseline visit between December 2006 and March 2009, a slightly different questionnaire was used to measure alcohol consumption (Table S20). We calculated daily alcohol intake in different types of drinks. The formulas were as follows: (1) Daily beer intakes (grams) = frequency * amount * weighted (different type of beer) * alcohol/unit; (2) Daily wine intakes (grams) = frequency * amount * weighted (different type of wine) * alcohol/ glass; (3) Daily distilled wine intakes (grams) = frequency * amount * alcohol/glass; (4) Daily alcohol intake (grams) = Daily beer intake + Daily wine intake + Daily distilled wine intake.

**Table S20. The items for alcohol use measured between December 2006 and March 2009**

| Items | Questions | Answers |
| --- | --- | --- |
| Food27 | How often did you drink alcoholic drinks in the past month? This includes non-alcoholic beer | “never”=0  “1 day a month”=1/28,  “2-3 days a month”=2.5/28,  “1 day a week”=1/7,  “2-3 days a week”=2.5/7, “4-5 days a week”=4.5/7, “6-7 days a week”=6.5/7. |
| Food28A | If you have drunk alcoholic beverages in the past month, how often did you drink a bottle/can of beer? | “never”=0%, “sometimes”=33%, “often”=67% ,  “always”=100%. |
| Food28B | If you have drunk alcoholic beverages in the past month, how often did you drink a swing top cap bottle/pint of beer? |
| Food28C | If you have drunk alcoholic beverages in the past month, how often did you drink a glass of beer? |
| Food29 | On days that you drank beer, how many glasses/bottles/cans did you drink on average? |  |
| Food30 | How often did you drink wine, sherry, port wine or vermouth in the past month? | “never”=0  “1 day a month”=1/28,  “2-3 days a month”=2.5/28,  “1 day a week”=1/7,  “2-3 days a week”=2.5/7, “4-5 days a week”=4.5/7, “6-7 days a week”=6.5/7. |
| Food31 | On days that you drank wine, sherry, port wine or vermouth, how many glasses did you drink on average? |  |
| Food32A | If you have drunk wine, sherry, port wine or vermouth in the past month, how often did you drink red wine or red port? | “never”=0%, “sometimes”=33%, “often”=67% ,  “always”=100%. |
| Food32B | If you have drunk wine, sherry, port wine or vermouth in the past month, how often did you drink other types of wine? |
| Food33 | How often did you drink distilled beverages such as genever, whisky, rum, gin, cognac, vieux, liqueur in the past month? | “never”=0  “1 day a month”=1/28,  “2-3 days a month”=2.5/28,  “1 day a week”=1/7,  “2-3 days a week”=2.5/7, “4-5 days a week”=4.5/7, “6-7 days a week”=6.5/7. |
| Food34 | On days that you drank distilled beverages such as genever, whisky, rum, gin, cognac, vieux, liqueur in the past month, how many glasses did you drink on average? |  |

For children aged 13-17 years, the FFQ [7], the self-report YSR [3] and the parent-report CBCL [2] were used. Current drinking was defined by combining the self-report questions “How often did you drink alcoholic beverages in the past month?”, “I drink alcohol without my parents’ permission now or in the past 6 months” and “How often did you drink alcoholic drinks in the past four weeks?” and parent-report question “To what extent does this description apply to your child now or in the past 6 months: drinks alcohol?” If one of the answers on these questions was “Yes”, current drinking was coded as “Yes”, otherwise it was coded as “No”. Alcohol consumption as a continuous phenotype was measured by the same FFQ as was used for adults recruited after March 2009. Daily alcohol intake was calculated by the same formula developed by WUR.

For children aged 8-12 years, information on alcohol use was collected by the parent-report CBCL [2]. Current drinking was defined by using the parent-report question “To what extent does this description apply to your child now or in the past 6 months: drinks alcohol?” If the answer was“a little bit or sometimes” or “clearly or often”, current drinking was coded as “Yes”, otherwise it was coded as “No”. Alcohol consumption as a continuous phenotype was not measured among children aged 8-12 years. We used the same question as a proxy for one child whose parents reported: “a little bit or sometimes” (all other children did not drink alcohol as reported by their parents). For this child, we used the median of daily alcohol intake for children aged 13-17 years who had the same answer to this question (2.53 grams/day, n=1).

**(5) Drug use**

For adults, drug use was measured at the second assessments by a self-report questionnaire [6]. Drug use was defined by using the question “Have you ever used any of the drugs listed below (cannabis, amphetamines, cocaine, heroin, magic mushrooms, ecstasy and other drugs)? (Yes/No)” Drug use frequency was defined using the question “How often did you use drugs in your entire life (cannabis, amphetamines, cocaine, heroin, magic mushrooms, ecstasy and other drugs)?” Based on the Dutch legal situation (i.e., the “Opium Act”^8^), we defined cannabis and magic mushrooms as soft drugs, and amphetamine, cocaine, heroin, and ecstasy as hard drugs.

For children aged 13-17 years, drug use was measured at baseline with parent-report using the CBCL [2], and self-report using the YSR [3]. Drug use was defined by combining self-report questions “Did you ever use drugs?” and “I use drugs now/in the past 6 months” and parent-report questions “Has your child ever used drugs?” and “To what extent does this description apply to your child now or in the past 6 months: uses drug?” If one of the answers on these questions was “Yes”, ever drug use was coded as “Yes”, otherwise it was coded as “No”. Drug use frequency was defined by using the question “How often in your life have you used drugs (cannabis, cocaine, amphetamines, heroin, magic mushrooms and other drugs)?”

For children aged 8-12 years, data on drug use was collected at baseline by the parent-reported CBCL [2]. Drug use was defined by using the parent-report question “To what extent does this description apply to your child now or in the past 6 months: uses drug?” If the answer was“a little bit or sometimes” or “clearly or often”, current drinking was coded as “Yes”, otherwise it was coded as “No”. As drug use frequency was not directly measured for children aged 8-12 years, we used the lowest 25 percentiles of drug use frequency of children aged 13-17 years who ever use drugs (1.5 times, n=2).

**Statistical Analysis**

**Recurrence risk ratio**

Analyses of familial aggregation of the same phenotype and co-aggregation between different phenotypes were performed for our dichotomous outcome measures in R3.5.2 (R; Vienna, Austria, 2013) [9]. The recurrence risk ratio (λ_R_) was calculated as the ratio between the prevalence of first-degree relatives of participants with the disease under study and its prevalence among the total Lifelines population [10]. We used a Cox proportional hazards model, adapted according to Breslow [11], to estimate λ_R_ in a cross-sectional study by applying an equal follow-up time for all participants. A marginal proportional hazards model was used to handle the correlation between observations due to familial clustering [12].

**Heritability and genetic correlation**

Univariate and bivariate quantitative genetic analyses were performed for continuous phenotypes, using ASReml 4.2 (ASReml; UK, 2016) [13]. ASReml fits linear mixed models using Restricted Maximum Likelihood to estimate variance components.

*Heritability estimate*: For the univariate analyses, we assumed the following linear mixed model for heritability estimation: **y**=**Xb** + **Z_a_a** + **e**,

where **y** is a vector of the response variable (e.g, BMI or depression), **b** is the vector of regression coefficients for the fixed effects, **a** are the additive genetic effects with variance σ^2^_a_**A**, and **e** are the residuals (environmental effects) with variance σ^2^_e_**I**. **X** is the design matrix of the fixed effects, **Z_a_** is the design matrix mapping subjects to the genetic kinship (relationship) matrix **A**. Narrow-sense heritability is defined as the proportion of phenotypic variance attributable to additive genetic variance [14], which was calculated as, *h*^2^= σ^2^_a_/(σ^2^_a_+σ^2^_e_), where σ^2^_a_ is additive genetic variance and σ^2^_e_ is the residual variance. Singletons in Lifelines were included in the analysis and contributed to the estimations of variances and phenotypic correlations but not to the genetic correlations.

*Genetic correlation estimate:* The genetic correlations were estimated using an expanded set of equations (i.e. a bivariate model) compared to the univariate analyses used to estimate heritability outlined above. In these bivariate analyses, the genetic correlations between two phenotypes were obtained from the estimated additive genetic covariance and variance components as:
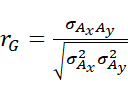
 , where σA_x_A_y_ is the additive genetic covariance between trait x and trait y and σ^2^A_x_, and σ^2^A_y_ is the additive genetic variance for traits x and y, respectively.

To test the significance of the heritability estimate (h^2^>0) and the genetic correlations (|r_G_|>0), the model in which all variances and correlations were estimated was compared to a model in which additive genetic variances and genetic correlations for all phenotypes were constrained to be zero, using a Likelihood Ratio Test. Similar analyses were conducted to test the significance of phenotypic correlations (|r_P_|>0), and environmental correlations (|r_E_|>0). Likewise, genetic correlations were constrained to be equal to 1 or -1 to test the presence of complete overlap of genetic effects (|r_G_|=1) [15].

**Reference**
